# Ecological Processes Shaping Marine Microbial Assemblages Diverge Between Equatorial and Temperate Time‐Series

**DOI:** 10.1111/mec.70241

**Published:** 2026-01-16

**Authors:** Pedro C. Junger, Vinicius S. Kavagutti, Ina M. Deutschmann, Carlota R. Gazulla, Paula Huber, Maiara Menezes, Rodolfo Paranhos, André M. Amado, Isabel Ferrera, Janaina Rigonato, Samuel Chaffron, Josep M. Gasol, Ramiro Logares, Hugo Sarmento

**Affiliations:** ^1^ Laboratory of Microbial Processes & Biodiversity, Departamento de Hidrobiologia Universidade Federal de São Carlos (UFSCar) São Carlos São Paulo Brazil; ^2^ Programa de Pós‐Graduação em Ecologia e Recursos Naturais, Centro de Ciências Biológicas e da Saúde Universidade Federal de São Carlos (UFSCar) São Carlos São Paulo Brazil; ^3^ Institut de Ciències del Mar (ICM) CSIC Barcelona Catalunya Spain; ^4^ CNRS, Laboratoire d'Ecogéochimie des Environnements Benthiques (LECOB), Observatoire Océanologique de Banyuls Sorbonne Université Banyuls‐sur‐Mer France; ^5^ Laboratorio de Microbiologia, Instituto Nacional de Limnología (INALI), CONICET‐UNL Ciudad Universitaria, Paraje El Pozo Santa Fé Argentina; ^6^ Departamento de Oceanografia e Limnologia Universidade Federal do Rio Grande do Norte (UFRN) Natal Rio Grande do Norte Brazil; ^7^ Instituto de Biologia Universidade Federal do Rio de Janeiro (UFRJ) Rio de Janeiro Rio Grande do Norte Brazil; ^8^ Departamento de Engenharia Sanitária e Ambiental Universidade Federal de Juiz de Fora (UFJF) Juiz de Fora Minas Gerais Brazil; ^9^ Centro Oceanográfico de Málaga Instituto Español de Oceanografía (IEO‐CSIC) Málaga Spain; ^10^ Génomique Métabolique, Genoscope, Institut François Jacob, CEA, CNRS, Univ Evry Université Paris‐Saclay Evry France; ^11^ Nantes Université École Centrale Nantes, CNRS, LS2N, UMR 6004 Nantes France; ^12^ Research Federation for the Study of Global Ocean Systems Ecology and Evolution FR2022/Tara Oceans GOSEE Paris France

**Keywords:** equatorial Atlantic, marine microbiome, Mediterranean Sea, metabarcoding, microbial observatory, network analysis

## Abstract

Marine microbial communities are structured by a complex interplay of deterministic and stochastic processes, yet how these vary across latitudes remains poorly understood. Most long‐term microbial observatories are restricted to temperate regions, limiting our ability to assess latitudinal contrasts in microbial dynamics. Here, we compare coastal microbial communities from two contrasting marine time‐series stations using standardised molecular protocols: a new tropical site in the Equatorial Atlantic (EAMO, 6° S) and a well‐studied temperate site in the Mediterranean Sea (BBMO, 41° N). Monthly 16S and 18S rRNA gene sequencing of two size‐fractions (0.22–3 μm and > 3 μm) over 41 months (from April 2013 to August 2016) revealed marked differences in taxonomic composition, temporal variability and ecological assembly processes. Temperate communities exhibited strong seasonal turnover, higher beta‐diversity and tighter coupling with environmental variables such as temperature and daylength. In contrast, tropical communities were compositionally more stable and more governed by biotic factors and stochastic processes such as historical contingency and ecological drift. These patterns were consistent across taxonomic domains and size‐fractions, though selection was generally stronger in prokaryotes and the smallest size‐fraction. Co‐occurrence networks at the temperate site were more densely connected and environmentally responsive compared to tropical networks, where stochastic processes and putative biological interactions gain prominence. This study highlights the importance of integrating observatories from underrepresented latitudes into global microbial monitoring efforts, particularly as climate change alters the amplitude and frequency of environmental drivers across the ocean.

## 1 Introduction

Marine microbial communities constitute a substantial component of Earth's biodiversity (de Vargas et al. 2015; Locey and Lennon 2016; McNichol et al. 2025), and their interactions are fundamental for Earth's ecosystem functioning and biogeochemical cycles (Falkowski et al. 2008; Guidi et al. 2016). Understanding the factors and mechanisms that drive microbial dynamics is important to predict how ongoing environmental changes impact their community composition, interactions and functions (Chaffron et al. 2021; Doney et al. 2012). Global oceanographic expeditions (e.g., *Tara* Oceans and Malaspina) and ocean‐basin transects (e.g., BioGEOTRACES and Bio‐GO‐SHIP) have revealed large‐scale spatial patterns of microbial diversity (Giner et al. 2020; Ibarbalz et al. 2019; McNichol et al. 2025; Salazar et al. 2016; Sunagawa et al. 2015) and their ecological associations (Chaffron et al. 2021; Deutschmann et al. 2024). The ecological processes shaping microbial communities have been shown to change between biological domains (prokaryotes vs. eukaryotes), as well as with ocean depth and spatial scale (Junger et al. 2023; Logares et al. 2020). However, these space‐for‐time studies lack the temporal dimension, which is essential to fully understand the patterns of microbial diversity and the underlying mechanisms shaping ocean microbial communities (Buttigieg et al. 2018; Moreira and López‐García 2019).

Most long‐term microbial observatories that have collected molecular data remain concentrated in northern mid‐latitudes (~22° to 50°) and often lack methodological standardisation (e.g., primer pairs, sequencing technology), limiting their inter‐comparison (Buttigieg et al. 2018; Raes et al. 2024). Meanwhile, most of the world's ocean surface is under warm oligotrophic tropical conditions (Behrenfeld et al. 2006), where primary production is usually dominated by picoplankton (< 2 μm), including prokaryotes (*Synechococcus* and *Prochlorococcus*) and small single‐cell eukaryotes (Li et al. 1983; Platt et al. 1983; Tarran et al. 2006). In this context, comprehensive comparative studies of ocean time‐series using standardised methods to assess microbial diversity and associations in the low‐latitude regions remain scarce, especially considering understudied areas such as the South Atlantic Ocean (Sarmento et al. 2025). There are some remarkable exceptions, though, such as the Australian Microbiome Initiative, that generated a dataset—conceived with standard methods and protocols—of seven coastal microbial time‐series covering a latitudinal gradient (~12° to 42° S) at continental scale (Brown et al. 2018). More recently, among these time‐series, the tropical Yongala observatory (~19° S) was shown to exhibit weak seasonal recurrence and increasing community dissimilarity over time, in contrast to several higher‐latitude sites, suggesting more stability and drift in tropical marine microbial communities (Raes et al. 2024). However, the ecological mechanisms underlying these observations in the tropical ocean remain poorly studied, particularly at equatorial latitudes (Chénard et al. 2019).

Efforts using ecological models have paved the way to understanding the ecological processes (selection, dispersal and drift) structuring microbial communities (e.g., Guo et al. 2024; Milke et al. 2022, 2023; Stegen et al. 2013; Vass et al. 2020; Wang et al. 2024). We have come to learn that prokaryotic community structures are relatively better explained by environmental selection than by dispersal or drift, while single‐cell eukaryotes' communities are more determined by the latter factors (Junger et al. 2023; Logares et al. 2020; Vass et al. 2020), mainly due to differences in organism and population sizes (Bie et al. 2012; Fodelianakis et al. 2021; Villarino et al. 2018), as well as the dormancy capacity of bacteria (Locey et al. 2020). The relative importance of these mechanisms changes with ocean depth due to differences in microbial abundances, environmental heterogeneity and barriers to dispersal (Junger et al. 2023). However, although biotic interactions are a fundamental component of deterministic processes in ecological theory (Vellend 2016), these ecological models rarely take them into account. As an alternative, co‐occurrence network topological metrics have been used to capture the ecological characteristics of plankton communities (Fuhrman et al. 2015), to investigate community resilience (Moore et al. 2016; Solé and Montoya 2001), as well as their potential responses to environmental changes (Chaffron et al. 2021). The application of network approaches to omics data has improved our understanding of potential ecological interactions between microbes, the role of keystone species and the likely response of these communities to ocean environmental changes (Chaffron et al. 2021; Cram et al. 2015; Deutschmann et al. 2021; Krabberød et al. 2022; Lima‐Mendez et al. 2015).

Here we compare the composition, stability and diversity of microbial communities—both prokaryotes and eukaryotes—in two coastal marine time‐series located at contrasting latitudes. We also aimed to compare the ecological processes, the environmental drivers and the microbial co‐occurrence network metrics between both sites. We determined which processes (deterministic selection vs. stochastic historical contingency and drift) temporally structure free‐living (0.22–3 μm) and particle‐associated (> 3 μm) prokaryotes as well as small (< 3 μm) and large (> 3 μm) protists in each time‐series. To do so, we sequenced 16S and 18S rRNA gene amplicons from DNA samples collected monthly during 41 months from April 2013 to August 2016 in two microbial observatories, one tropical site located in the Western Equatorial Atlantic (6° S) and one temperate site located in the Northwestern Mediterranean Sea (42° N). Tropical zones, which have limited variation in daylength and temperature over time, may represent a case of weak abiotic selection, while in temperate zones, abiotic selection is expected to change with seasons. As a consequence, tropical microbial communities should be more stable over time when compared to those of temperate regions, and historical and drift‐like stochastic processes may arise as the main drivers structuring these communities. In this sense, we hypothesize that temperate microbial communities are primarily driven by abiotic selection caused by the seasonal fluctuation of physical variables. Meanwhile, in tropical regions where daylength and temperature remain nearly constant year‐round, selection is exerted predominantly by multiple biotic factors.

## 2 Material and Methods

### 2.1 Study Sites and Sampling Design

**FIGURE 1:**
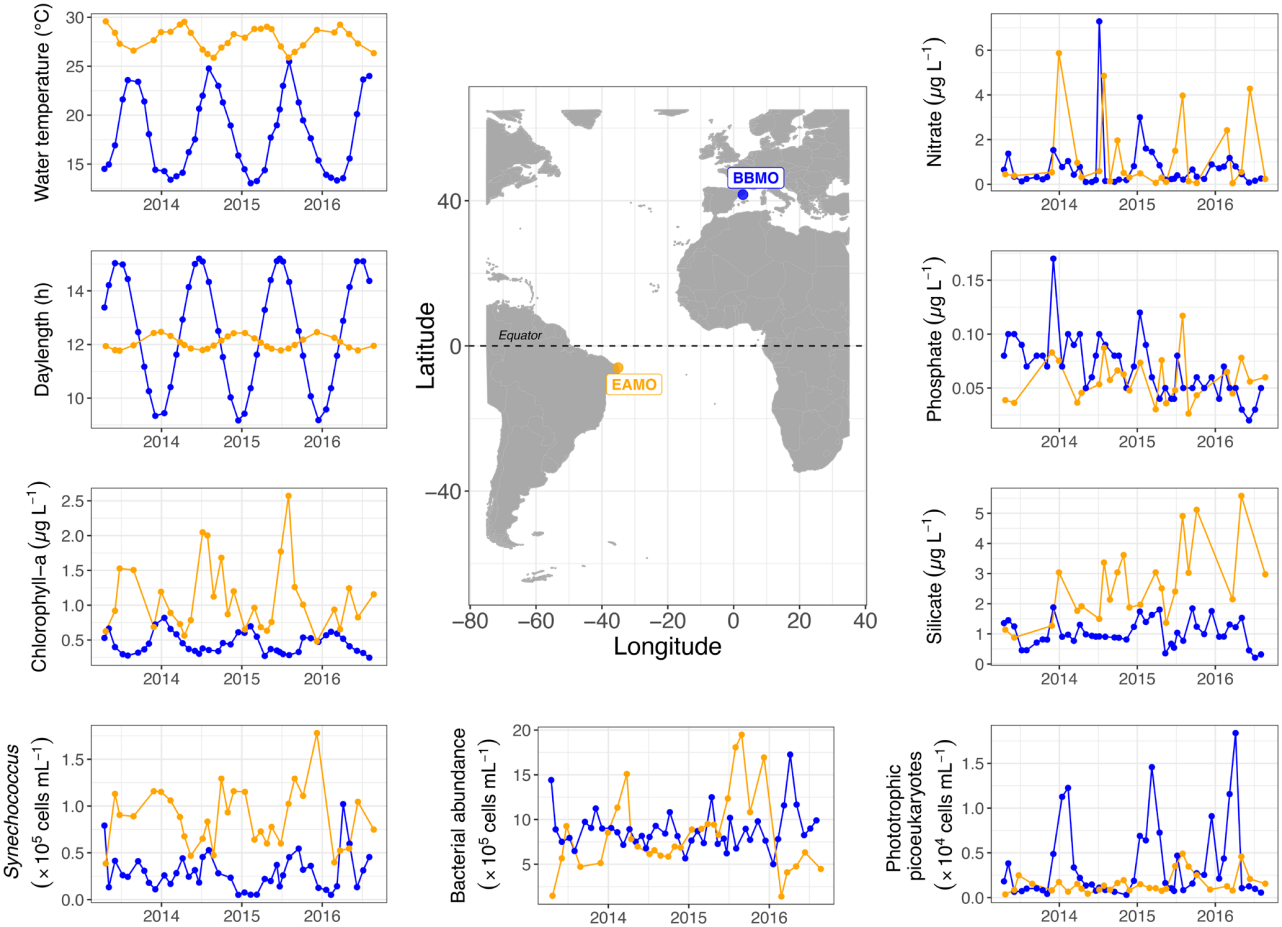
The geographic location of the two contrasting coastal marine microbial observatories sampled in this study: The Equatorial Atlantic Microbial Observatory (EAMO, 6° S) and the Blanes Bay Microbial Observatory (BBMO, 42° N). Temporal variability in temperature, daylength, chlorophyll‐a, inorganic nutrients and microbial abundances in these observatories during the sampling period of this study (April 2013–August 2016). Figure S1 shows the differences between observatories for all measured variables.

Surface seawater (~1 m depth) samples were collected monthly from April 2013 to August 2016 at two coastal marine observatories: the Equatorial Atlantic Microbial Observatory—EAMO (−5.99°, −35.08°) located at the western coast of the Atlantic, 30 km away from the city of Natal (Brazil); and the LTER Blanes Bay Microbial Observatory—BBMO (41.66°, 2.80°) located in the Northwestern Mediterranean Sea (Figure 1). The BBMO is a well‐studied temperate oligotrophic coastal site ~1 km offshore with little riverine or human influence (Gasol et al. 2016), while the EAMO is a newly established tropical oligotrophic site ~3 km from the Brazilian coastline. Both coastal sites are on average 20 m deep. To our knowledge, the EAMO is the first microbial observatory located in the South Atlantic Ocean (Menezes et al. 2023), and one of the very few observatories in low latitudes (0°–10°) with available amplicon sequencing data. On the other hand, the BBMO is a long‐standing microbial observatory with several publications using amplicon sequencing data (e.g., Auladell et al. 2022; Ferrera et al. 2024; Giner et al. 2019). These observatories have contrasting variation in environmental characteristics, notably temperature and daylength (Figure 1), and significant differences in most environmental variables (Figure S1).

At each site, 20 L sub‐surface (~1 m depth) seawater samples were passed through a 200‐μm mesh net and transported to the lab in 20‐L polycarbonate carboys, under dim light and processed within 1.5 h. To obtain microbial biomass, 2–6 L of surface seawater was first filtered through a 20‐μm filter nylon mesh and then sequentially filtered through 3 μm polycarbonate filters (hereafter ‘> 3 μm’ size‐fraction; ‘particle‐associated prokaryotes’ for prokaryotes associated with particles or eukaryotic organisms, and ‘nanoeukaryotes’ for large eukaryotes) and 0.22 μm Sterivex ‘cartridges’ (Millipore) (hereafter ‘0.22–3 μm’ size‐fraction; ‘free‐living’ for prokaryotes and ‘picoeukaryotes’ for small eukaryotes), using a peristaltic pump. The filters were embedded in a lysis buffer solution and maintained frozen (−80°C) until DNA extraction.

Daylength (hours of light) was calculated for both sites based on coordinates and sampling dates. The accumulated rainfall data (Figure S2) was obtained from the closest meteorological stations through the National Institute of Meteorology (*INMET*, Brazil) for EAMO, and through the Catalan Meteorological service (www.meteo.cat) for the BBMO. To evaluate the potential influence of inter‐annual changes on community composition in the EAMO and the BBMO, monthly values of the Southern Oscillation Index (SOI) and the Western Mediterranean Oscillation Index (WeMOI) were obtained from the NOAA database (https://www.ncei.noaa.gov/access/monitoring/enso/soi) and the University of Barcelona Climatology Group database (Lopez‐Bustins et al. 2020), respectively (Figure S2).

### 2.2 Analytical Methods

Water transparency was determined with a Secchi disk. Water temperature and salinity were measured in situ with a multiparameter probe (Horiba U‐50 Series) in the EAMO, and a CTD model SAIV A/S SD204 in the BBMO. In situ chlorophyll‐a was measured at the BBMO (not measured at the EAMO) by filtering the seawater in 3 μm polycarbonate filters and then GF/F (Whatman), and extracted with acetone (90% acetone, 4°C, overnight), and determined by fluorometry (Yentsch and Menzel 1963). Satellite‐based chlorophyll‐a estimates were obtained from the ESA OceanColour‐CCI database (OC‐CCI5; https://www.oceancolour.org/) for both sites with a daily 3 × 3 pixel box. Then, a 30‐day moving average was calculated for each sampling date of both sites. Since satellite and in situ chlorophyll‐a estimates were strongly correlated (Pearson's *r* = 0.78, *p* < 0.01) at the BBMO (Figure S3), the satellite‐derived chlorophyll‐a was used as a standard comparative proxy of chlorophyll‐a between the observatories. Dissolved inorganic nutrient (NO_3_^−^, NO_2_^−^, NH_4_^+^, PO_4_^3−^, SiO_2_) concentrations were determined from GF/F (Whatman) filtered seawater samples in an autoanalyzer following standard procedures (Grasshoff et al. 2009).

Seawater samples for flow cytometry counts were preserved with 1% paraformaldehyde +0.05% glutaraldehyde (final concentration). Bacterial abundance was determined in a BD FACSCalibur flow cytometer with a blue (488 nm) laser and SybrGreen I staining, according to (Gasol and del Giorgio 2000). Picocyanobacteria were subtracted from independent counts of non‐stained samples in a plot of side light scatter (SSC) versus red (FL3) and orange (FL2) fluorescences. Phototrophic picoeukaryotes were quantified from 400 μL non‐stained samples run at high speed (ca. 52.3 μL min^−1^) using SSC, FL3 and FL2 cytometric signatures (Menezes et al. 2023). Flow cytometric data acquisition and analysis were performed with the FlowJo V10.0.8 software. Bacterial production rates were measured using the [^3^H]‐leucine incorporation method (Kirchman 1992). Further details on the analytical methods are provided in the Supporting Information.

### 2.3 DNA Extraction, Sequencing and Bioinformatics

DNA extraction was carried out with a phenol‐chloroform protocol (Logares et al. 2014), and subsequent purification using Amicon columns (Millipore 100KDa/100.000MWCO), following the manufacturer's instructions. DNA extracts were quantified with a Qubit 1.0 (Thermo Fisher Scientific) and preserved at −80°C. PCR amplification was performed using the primers 515F‐Y (5′‐GTGYCAGCMGCCGCGGTAA) and 926R (5′‐CCGYCAATTYMTTTRAGTTT) for the 16S rRNA gene hypervariable V4‐V5 region (≈400 bp) to target prokaryotes—both Bacteria and Archaea (Parada et al. 2016). For prokaryotes, a mock community with 25 bacterial and 2 archaeal strains (Supporting Information, Table S1) was included as a positive control in the sequencing batch. Taxa included in the mock community were consistently recovered in the sequencing results (Figure S4), confirming primer compatibility and sequencing reliability. For eukaryotes, the primers used were TAReukFWD1 (5′‐CCAGCASCYGCGGTAATTCC‐3′) and TAReukREV3 (5′‐ACTTTCGTTCTTGATYRA‐3′) of the 18S rRNA gene hypervariable V4 region (≈380 bp) (Stoeck et al. 2010). No mock community was used for the 18S rRNA gene dataset, as generating representative protist mocks remains technically challenging (e.g., Catlett et al. 2020; Marinchel et al. 2023) and beyond the scope of this study (Supporting Information). Negative controls (extraction and PCR blanks) were included in both sequencing batches.

Samples were sequenced in an Illumina MiSeq platform (2 × 300 bp) in two different sequencing runs (Supporting Information). Raw reads were processed using DADA2 (Callahan et al. 2016) to determine amplicon sequence variants (ASVs). For the 16S rRNA gene, we trimmed the forward reads at 210 bp, and the reverse reads at 180 bp, while for the 18S rRNA gene, forward reads were trimmed at 220 bp and the reverse reads at 190 bp. Afterwards, for the 16S rRNA gene, the maximum number of expected errors (maxEE) was set to 5 for the forward and reverse reads, while for the 18S rRNA gene, the maxEE was set to 5 and 6 for the forward and reverse reads, respectively. Finally, error rates were estimated using DADA2 for both the 16S and 18S genes to delineate the ASVs.

ASVs taxonomy was assigned with DADA2 using the naïve Bayesian classifier method (Wang et al. 2007) together with the SILVA v.138 database (Quast et al. 2013) for prokaryotes, and the Protist Ribosomal Reference database (PR^2^, version 4.14, (Guillou et al. 2013)), for eukaryotes. Eukaryotes, chloroplasts and mitochondria were removed from the 16S ASVs table, while Holozoan (Metazoa and Fungi), Streptophyta and nucleomorphs were removed from the 18S ASVs table. A total of 4,922,683 reads were obtained for the 16S rRNA and 18,844,773 reads for the 18S rRNA after quality filtering and removal of the above‐mentioned sequences. The samples' prokaryotic and eukaryotic taxonomic compositions, segregated both by observatory and size‐fraction, are presented in the Supporting Information (Figure S5). For ecological models and statistical analyses, the prokaryotic and eukaryotic ASV tables were rarefied down to 8063 and 9513 reads per sample, respectively, using the *rrarefy* function from the *vegan* R package (Oksanen et al. 2024). For network analyses, the prokaryotic and eukaryotic read abundance tables were not rarefied but were separately subjected to a centered‐log‐ratio transformation before being merged to account for data compositionality (Gloor et al. 2017).

### 2.4 General Statistical Analyses

We used the R software v 4.0.3 (R Core Team 2013) with the packages *vegan* (Oksanen et al. 2024) and *BiodiversityR* (Kindt and Coe 2005) for data processing and statistical analyses. In order to detect differences in community composition between sites, Bray–Curtis dissimilarity distances between sites using ASVs' relative abundances were calculated and plotted in a non‐metric multidimensional scaling plot using the *vegan* package in R. Statistical differences between sites, size‐fractions and seasons were tested by Permutational MANOVA (PERMANOVA) analysis with the ‘adonis2()’ function in the *vegan* package. Analyses of dissimilarities were also conducted using the ‘adonis2()’ function of the *vegan* R package to investigate the percentage of variance in community composition explained by biological (prokaryotic vs. eukaryotic community dissimilarity matrices) and environmental variables (McArdle and Anderson 2001).

### 2.5 Defining Common and Indicator ASVs

Shared and unique ASVs were found with Venn plots using the *ggvenn* R package. We categorised the shared ASVs with more than 50% occurrence in both sites as ‘common’ ASVs. Since some shared ASVs were significantly more abundant in a given site, we carried out a taxa indicator analysis to identify ASVs that were not necessarily unique, but were strongly associated to a given site/size‐fraction (Cáceres and Legendre 2009). We performed this indicator analysis using the ‘multipatt()’ function of the R package *indicspecies* v.1.7.8 with parameter ‘IndVal.g.’, with an ASV considered as an indicator of a given site/size‐fraction when the association value was > 0.7 and the *p*‐value was < 0.01. The ASVs only present in the EAMO and the BBMO were called ‘EA‐exclusive’ and ‘BB‐exclusive’, respectively. The remaining ASVs that did not fall into any of the above categories were classified as ‘background’.

### 2.6 Determining Seasonal ASVs

Seasonality was evaluated by testing the occurrence of periodic patterns in the ASVs from both the 16S and 18S rRNA gene datasets, at each size‐fraction and site. We used the Lomb Scargle periodogram (LSP) implemented in the *lomb* v.2.5.0 R package, as it has been successfully applied in previous studies with uneven sampled signals (Auladell et al. 2022; Ferrera et al. 2024; Lambert et al. 2019; Ruf 1999). For each dataset, we analysed the ASVs appearing in at least 10% of samples and applied the LSP separately using the ‘randlsp()’ function with parameters: normalise = ‘standard’ and type = ‘period’. After a manual inspection (Supporting Information), we considered an ASV as seasonal when it presented a PN > 0.2, a *p*‐value < 0.01 and an interval of ~1 year.

### 2.7 Computation of Ecological Processes

Phylogenetic trees were built for both the 16S and 18S rRNA gene datasets to obtain the phylogenetic distances for the ecological model (see section below). First, we aligned raw ASV sequences against an aligned SILVA template—for 16S rRNA—and an aligned PR^2^ template—for 18S rRNA—using mothur (Schloss et al. 2009). Poorly aligned regions or sequences were removed using *trimAl* (parameters: ‐gt 0.3 ‐st 0.001) (Capella‐Gutiérrez et al. 2009). The alignment was visually curated with *seaview* v4 (Gouy et al. 2010), and sequences with ≥ 40% of gaps were removed. Phylogenetic trees were inferred from the curated alignment using FastTree v2.1.9 (Price et al. 2009).

We estimated the relative importance of ecological processes shaping microbial communities using a null model approach (Stegen et al. 2013) that has been widely used in ecological studies (Gazulla et al. 2022; Huber et al. 2020; Junger et al. 2023; Logares et al. 2020; Vass et al. 2020). This analysis consists of inferring environmental selection from ASV phylogenetic turnover (Table 1, Figure S6). First, we determined the phylogenetic turnover using the abundance‐weighted β‐mean nearest taxon distance (βMNTD) metric (Stegen et al. 2013), which computes the mean phylogenetic distances between each ASV and its closest relative in each pair of communities (pairwise comparisons). Second, we ran null models with 999 randomisations to simulate the community turnover by chance (βMNTD_null_) (Stegen et al. 2013). Finally, the β‐Nearest Taxon Index (βNTI) was calculated from the differences between the observed βMNTD and the mean βMNTD_null_ values. Overall, |βNTI| > 2 indicates that taxa are phylogenetically more related or less related than expected by chance, pointing to a strong influence of selection on community assembly (Stegen et al. 2013). More precisely, βNTI values higher than 2 indicate the action of heterogeneous selection, while βNTI values lower than −2 point to the action of homogeneous selection (Stegen et al. 2013). The fraction of β‐diversity of the communities that was not explained by selection (|βNTI| ≤ 2) was assessed in a second step. This typically involves calculating ASV taxonomic turnover to evaluate the roles of dispersal and ecological drift in shaping community structure (Stegen et al. 2013). However, because dispersal cannot be directly inferred from temporal samples collected at a fixed site, we redefined dispersal as ‘non‐selective turnover’ (hereafter referred to as ‘non‐selection’), dispersal limitation as ‘historical contingency’ (Fukami 2015) and homogenising dispersal as ‘non‐selective low turnover’, adapted for a temporal framework (Table 1). We computed the Raup‐Crick metric (RCbray) based on Bray–Curtis dissimilarities (Chase and Myers 2011; Stegen et al. 2013). The RCbray metric compares observed β‐diversity to that derived from null models generated through 999 randomisations, representing random community assembly or ecological drift. RCbray values range from −1 to 1, with 0 indicating no deviation from the null expectation. A threshold of |RCbray| > 0.95 (two‐tailed test, *α* = 0.05) indicates that two communities differ significantly from the null model (Stegen et al. 2013). Values of |RCbray| ≤ 0.95 suggest community assembly driven solely by ecological drift (i.e., random processes) (Chase and Myers 2011), while RCbray > 0.95 or < −0.95 indicates that community assembly is structured by historical contingency or non‐selective low turnover, respectively (Table 1).

**TABLE 1:** Definition of ecological processes in a temporal framework (adapted from the spatial framework in Stegen et al. 2013). A schematic figure is available in the Supporting Information (Figure S6).

β‐diversity pattern	Spatial framework	Temporal framework	Interpretation	Thresholds
High, due to selection	Heterogeneous selection	Heterogeneous selection	Environmental conditions shift over time, selecting for different taxa, causing changes in community composition (e.g., environmental filtering)	βNTI > 2
Low, due to selection	Homogeneous selection	Homogeneous selection	Stable environment filtering maintaining similar taxa	βNTI < −2
High, not selection	Dispersal limitation	Historical contingency (or non‐selective high turnover)	Temporal divergence due to stochastic or historical events (e.g., priority effects, disturbances)	|βNTI| ≤ 2; RCbray > 0.95
Low, not selection	Homogenising dispersal	Non‐selective low turnover	Rare, persistent similarity not attributable to selection or ecological drift	|βNTI| ≤ 2; RCbray < −0.95
Within null expectation	Ecological drift	Ecological drift	Stochastic changes in growth‐death rates with no strong deterministic forces detected	|RCbray| ≤ 0.95

### 2.8 Network Construction

Microbial association networks consist of nodes representing microorganisms and edges indicating potential interactions. For network construction, we used only samples that had both 16S and 18S data and ‘small’ (0.22–3 μm) and ‘large’ (> 3 μm) size‐fractions (Table S2, Figure S7). First, to control for data compositionality in network construction (Gloor et al. 2017), we applied a centred‐log‐ratio transformation separately to the prokaryotic and protist ASV tables of both size‐fractions and locations. Second, we merged the four data tables corresponding to each primer/size‐fraction (16S 0.22–3 μm, 16S > 3 μm, 18S 0.22–3 μm and 18S > 3 μm) into a single matrix for each observatory. Then, we constructed one global network for each observatory (BBMO and EAMO) using FlashWeave (Tackmann et al. 2019), selecting the options ‘heterogeneous’ and ‘sensitive’. Next, we filtered environmentally driven edges via EnDED and edges with a co‐occurrence score below 50% (Deutschmann et al. 2021). Isolated nodes, that is, nodes without an edge, were removed. The resulting (static) BBMO network contained 1906 nodes and 2614 edges (2393 or 92% positive and 221 or 8% negative), while the (static) EAMO network contained 2074 nodes and 2389 edges (2068, 87% positive and 321, 13% negative) (Table S2). Finally, we approximated the temporal networks via monthly subnetworks (23 in the BBMO and 22 in the EAMO) from each static network (Deutschmann et al. 2023). Each temporal network contains a subset of nodes and edges of the global static network. An edge is present in a subnetwork of a particular month if it was present in the static network and both nodes correspond to microorganisms detected in that month. A microorganism is determined as detected if the sequence abundance is above zero.

### 2.9 Network Analysis

We computed global network metrics to characterise the single static network and each monthly subnetwork using the *igraph* R package (Csardi and Nepusz 2006) and adapted code from (Deutschmann et al. 2023, 2024). The computed metrics included edge density, average path length, transitivity, mean degree and assortativity based on node degree, domain (prokaryotes vs. protists) and size‐fraction (small vs. large). We also calculated the average strength of positive associations between nodes. The definitions and ecological interpretations of these metrics are summarised in Table 2. Spearman correlations between global network metrics and environmental data were computed using the *corr.test* function in the *psych* R package (Revelle 2025), with Holm's correction for multiple testing (Holm 1979). Network visualisations were generated with Gephi v.0.10.1 (Bastian et al. 2009) using the Fruchterman–Reingold layout (Area = 10,000; Gravity = 1; Speed = 10).

**TABLE 2:** Definition and ecological rationale of the network topological metrics computed in this study.

Topological metric	Definition	Ecological rationale
N° of nodes	Nodes represent microorganisms in the network	Species richness, after removing isolated nodes, representing the total number of unique species (ASVs) in the microbial network (Dunne et al. 2002; Röttjers and Faust 2018)
Edge density	Ratio between the number of edges and the number of possible edges. Measures how well the graph is connected	Connectivity index capturing the proportion of possible links between ASVs, indicating the extent of interactions or associations (Barberán et al. 2012; Deutschmann et al. 2023)
Mean positive strength	Mean of all positive association scores	Index of the strength of positive associations or co‐occurrences between microbial taxa (Deutschmann et al. 2023; Faust and Raes 2012)
Transitivity	Ratio of closed triplets to all triplets (closed and open), ranging from 0 to 1. Measures clustering, specifically, it measures the probability that two neighbours of a node are also connected	Captures the tendency of species that are connected to a common species, to also be connected, often reflecting ecological groupings or mutualistic interaction networks (Deutschmann et al. 2023; Newman 2002)
Average path length	Average length of all possible shortest paths in the graph	Shorter path lengths indicate that any two nodes are connected through relatively few intermediate links, suggesting a well‐connected community structure (Albert et al. 2000; Guo et al. 2022)
Assortativity degree	Quantifies whether nodes tend to connect to nodes with similar characteristics (here numerical ‘degree’)	Indicates network organisation and whether similarity in ecological characteristics is associated with preferential linking patterns in the community (Newman 2002; Röttjers and Faust 2018)
Assortativity (Prot vs. Prok)	Quantifies whether nodes tend to connect to nodes with similar characteristics (here categorical ‘domain’), that is, positive if protists connect with protists and prokaryotes with prokaryotes; negative if cross‐domain connections dominate	Provides insight into microbial community structure, whether different domains interact more or less with each other (Deutschmann et al. 2023, 2024)
Assortativity (Small vs. Large)	Quantifies whether nodes tend to connect to nodes with similar characteristics (here categorical ‘size‐fraction’), that is, positive if small‐fraction (0.22–3 μm) nodes connect with each other and large‐fraction (> 3 μm) nodes do likewise; negative if cross‐fraction connections dominate	Reveals whether microbial taxa in the small size fraction tend to form more associations with taxa in the same size‐fraction or across size‐fractions (Deutschmann et al. 2023, 2024)

## 3 Results

### 3.1 Equatorial Microbial Communities Exhibit Lower Turnover Than Those at the Temperate Observatory

**FIGURE 2:**
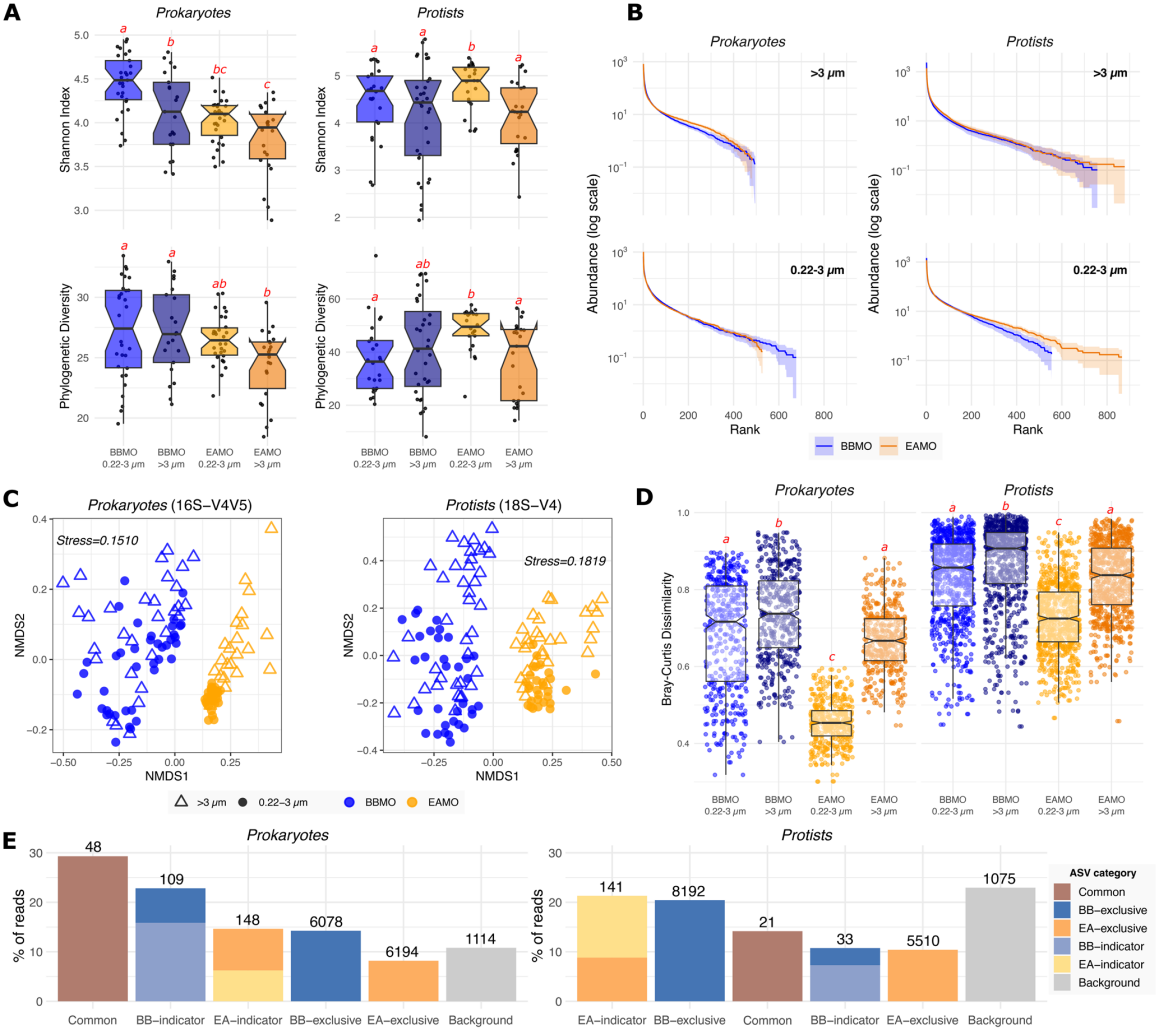
Differences in microbial community diversity between the temperate site (BBMO) and the tropical site (EAMO). (A) Shannon index and phylogenetic diversity (Figure S8 includes amplicon sequence variants (ASV) richness, and Pielou's evenness, while Figure S9 shows seasonal variability in diversity metrics). Different red letters represent significantly different means (ANOVA, Tukey post hoc test, *p* < 0.05); (B) Normalised rank abundance plots of the prokaryotic and protist communities. The line stands for the average rank abundance of each fraction and site. The area around the lines represents rank abundance dispersion of each sample subset; (C) Non‐metric multidimensional scaling (NMDS) based on the Bray–Curtis dissimilarities among prokaryotic and eukaryotic samples—labelled by observatory (colours) and size‐fraction (shapes); (D) Beta‐diversity between observatories and size‐fractions; (E) Percentage of reads of the ASV categories, as defined in the methods section. The number (#) of ASVs within each category is given above the bars. Note that some exclusive ASVs were also indicators for prokaryotes (*n* = 51 in BB; *n* = 91 in EA) and protists (*n* = 19 in BB; *n* = 80 in EA). BB, BBMO, Blanes Bay Microbial Observatory; EA, EAMO, Equatorial Atlantic Microbial Observatory.

Alpha diversity analyses revealed significantly lower prokaryotic diversity at the tropical site relative to the temperate site (Mann–Whitney Rank Sum test, *p* < 0.01; Figures 2A and S7). Prokaryotic compositional diversity was significantly higher in the free‐living (0.22–3 μm) than in the particle‐associated (> 3 μm) fraction at the BBMO (ANOVA, *p* < 0.001), but did not differ significantly between fractions at the EAMO. In contrast, small protist diversity was greater at the EAMO than at the BBMO (Mann–Whitney test, *p* < 0.001), although this pattern did not extend to larger protists (Figures 2A and S7). Seasonal variation in alpha diversity was evident at the BBMO but not at the EAMO (Figure S9). Prokaryotic diversity peaked during winter, while protist diversity was highest in autumn at the BBMO. Conversely, the EAMO displayed no significant seasonal fluctuations in alpha diversity, consistent with a more temporally stable tropical microbiome. Rank abundance curves further illustrated these differences: the BBMO communities were dominated by fewer, highly abundant ASVs, whereas the EAMO harboured a greater number of less abundant taxa, a pattern especially pronounced for protists (Figure 2B).

Beta‐diversity analyses showed strong segregation of microbial community composition by site (Figure 2C), which was a more informative factor than size‐fraction in explaining variability for both prokaryotic (PERMANOVA, site *R* ^2^ = 0.22, size‐fraction *R* ^2^ = 0.06, *p* < 0.001) and protist (site *R* ^2^ = 0.12, size‐fraction *R* ^2^ = 0.05, *p* < 0.001) communities. The equatorial site exhibited significantly lower temporal turnover (Bray–Curtis dissimilarity) in both domains and size‐fractions compared to the temperate site (Figure 2D). At BBMO, seasonality explained more variation than size‐fraction for both prokaryotic (PERMANOVA, season *R* ^2^ = 0.29, size‐fraction *R* ^2^ = 0.07, *p* < 0.001) and protist (season *R* ^2^ = 0.14, size‐fraction *R* ^2^ = 0.07, *p* < 0.001) communities (Figure S10). In contrast, at EAMO, seasonality accounted for less variation than size‐fraction in the prokaryotic community (season *R* ^2^ = 0.09, size‐fraction *R* ^2^ = 0.19, *p* < 0.001), whereas in the protist community, seasonality (*R* ^2^ = 0.11) and size‐fraction (*R* ^2^ = 0.08, *p* < 0.001) explained a similar fraction of the variation.

### 3.2 Higher Stability and Number of Microbial Indicators in the Equatorial Site

Despite a similar percentage of shared ASVs between biological domains and size‐fractions (~8%–9%; Figure S11), marked differences emerged in the distribution and identity of common and site‐specific indicator ASVs. Among shared particle‐associated prokaryotic ASVs (*n* = 788), 23 (~3%) were classified as common, while among free‐living prokaryotic ASVs (*n* = 703), 41 (~6%) were common, with 16 shared across both size‐fractions, 25 exclusive to the free‐living fraction and 7 to the particle‐associated fraction. For protists, common ASVs were rarer: 6 (~0.8%) of 768 ASVs in the large size‐fraction and 16 (~2%) of 832 ASVs in the small size fraction, with only two ASVs found to be common in both size‐fractions. The percentage of unique prokaryotic ASVs in each observatory was similar between size‐fractions, with ~46% in the BBMO and ~45% in the EAMO (Figure S11). In contrast, the BBMO harboured more unique protist ASVs than the EAMO in both fractions (small: 50% vs. 41%; large: 53% vs. 39%; Figures S11–13). The indicator species analysis revealed more indicator ASVs at EAMO (148 prokaryotes, 141 protists) than BBMO (109 and 33, respectively) (Figure 2E). Many site‐exclusive ASVs were also indicators: at EAMO, 91 prokaryotes (~61%) and 80 protists (~57%); at BBMO, 51 prokaryotes (~47%) and 19 protists (~58%).

**FIGURE 3:**
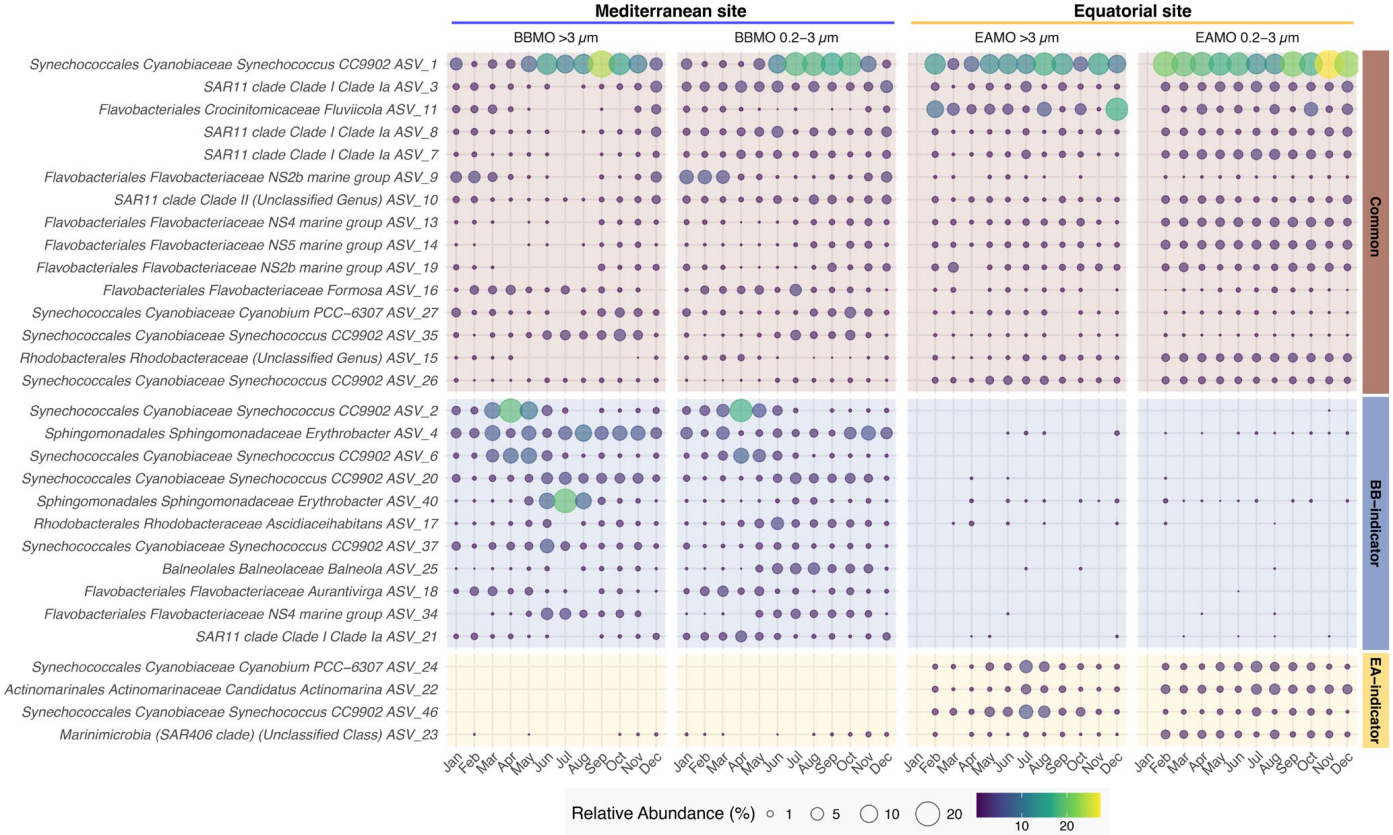
The monthly average relative abundance of the 30 most abundant prokaryotic ASVs classified as common, BB‐indicators or EA‐indicators. A version of this figure with the 100 most abundant prokaryotic ASVs is presented in the Supporting Information (Figure S14). ASV, amplicon sequence variants; BB, BBMO, Blanes Bay Microbial Observatory; EA, EAMO, Equatorial Atlantic Microbial Observatory.

The 30 most abundant prokaryotic ASVs highlighted contrasting temporal dynamics (Figure 3). At BBMO, strong seasonality and niche partitioning were evident for common and indicator taxa. For example, *Synechococcus* CC9902 common ASVs peaked in summer, while BBMO‐specific ASVs dominated in early spring; Flavobacteriaceae ASVs (e.g., NS4/NS5 groups, *Formosa*) increased in autumn/winter, and ASVs assigned to taxa such as *Balneola*, *Aurantivirga* and *Erythrobacter* were largely confined to the BBMO. In contrast, EAMO communities were more temporally stable, with common (*Synechococcus* CC9902, Flavobacteriaceae, SAR11, *Cyanobium* PCC‐6307) and site‐indicator (*Actinomarinales*, SAR406, *Cyanobium* PCC‐6307) ASVs showing little temporal change. An exception was a common ASV assigned to the genus *Fluviicola*, which exhibited more striking temporal variability in the larger size‐fraction at EAMO. Notably, ASV‐level niche partitioning was observed in *Synechococcus* CC9902, with some ASVs acting as generalists and others showing site‐specific dominance.

**FIGURE 4:**
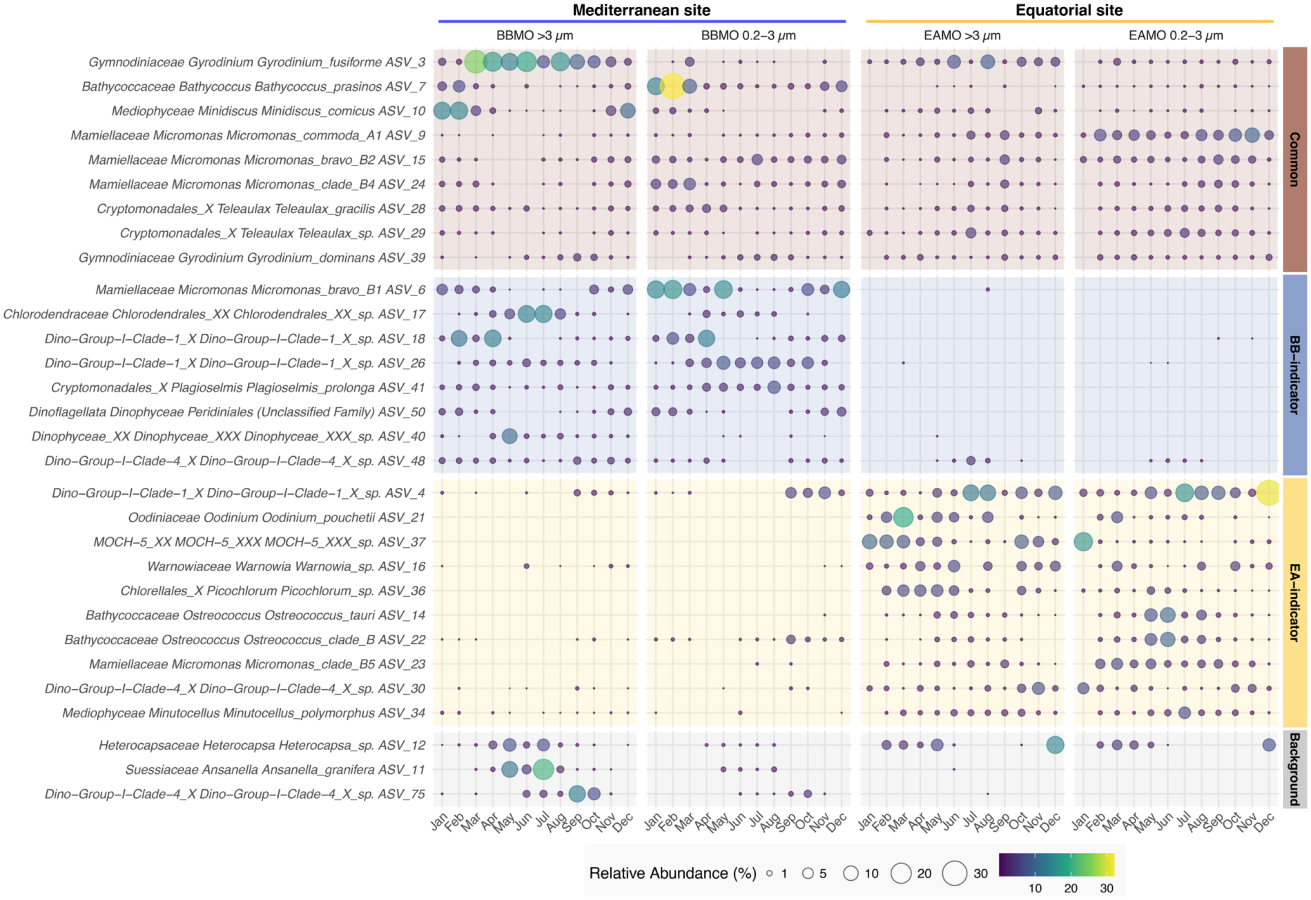
The monthly average relative abundance of the 30 most abundant protist ASVs classified as common, BB‐indicators, EA‐indicators or background. A version of this figure with the 100 most abundant protist ASVs is presented in the Supporting Information (Figure S15). ASV, amplicon sequence variants; BB, BBMO, Blanes Bay Microbial Observatory; EA, EAMO, Equatorial Atlantic Microbial Observatory.

The differences in seasonal and niche partitioning patterns between sites were even clearer in protists (Figure 4). At BBMO, distinct seasonal patterns and niche partitioning appeared among both common and indicator protist ASVs. For example, ASVs assigned to the common *Bathycoccus prasinos* and the BBMO‐indicator *Micromonas bravo* B1 peaked in winter and early spring, while other common ASVs assigned to the *Micromonas* clades (*M. bravo* B2 and *M*. clade B4) slightly increased during spring. The small centric diatom *Minidiscus comicus* was also more abundant in winter. Conversely, ASVs assigned to *Gyrodinium dominans* and background dinoflagellates like *Ansanella granifera* and *Heterocapsa* peaked in warmer months, likely associated with seasonal stratification and low nutrient availability. The cryptophyte *Teleaulax gracilis* had moderate abundance peaks in spring, while BBMO‐indicator ASVs assigned to the cryptophyte *Plagioselmis prolonga* and green algae *Chlorodendrales* increased in summer. Parasitic dinoflagellate (Syndiniales) ASVs from Dino‐Group‐I‐Clade‐1 showed temporal niche partitioning, with early and late spring peaks for different ASVs, whereas Dino‐Group‐I‐Clade‐4 increased in autumn.

Although less pronounced than at BBMO, protist community temporal fluctuations were still observed at the EAMO (Figure 4). Common *Micromonas* clades (*M. commoda* A1, *M. bravo* B2 and *M*. clade *B4*) as well as the EAMO‐indicator *Micromonas* clade B5 were present year‐round with moderate abundance and minimal fluctuation. In contrast, EAMO‐indicators *Ostreococcus tauri* and *Ostreococcus* clade B peaked mid‐year, coinciding with the region's rainy season (Figure S2). Common *Gyrodinium fusiforme* and *G. dominans* persisted across seasons with little variation. The small diatom *Minutocellus polymorphus* was consistently detected year‐round, with a mid‐year peak. Syndiniales parasites belonging to Dino‐Group‐I‐Clade‐1 and ‐Clade‐4, along with *Oodinium pouchetii* and the athecate dinoflagellate genus *Warnowia*, were also persistent throughout the year. Among EAMO‐indicators, ASVs assigned to the phototrophic Marine Ochrophyta clade 5 (MOCH‐5) and the green algae *Picochlorum* showed some temporal variation, with relative abundances dropping mid‐year.

### 3.3 Weaker Seasonal Patterns in the Equatorial Site Than in the Temperate Site

**FIGURE 5:**
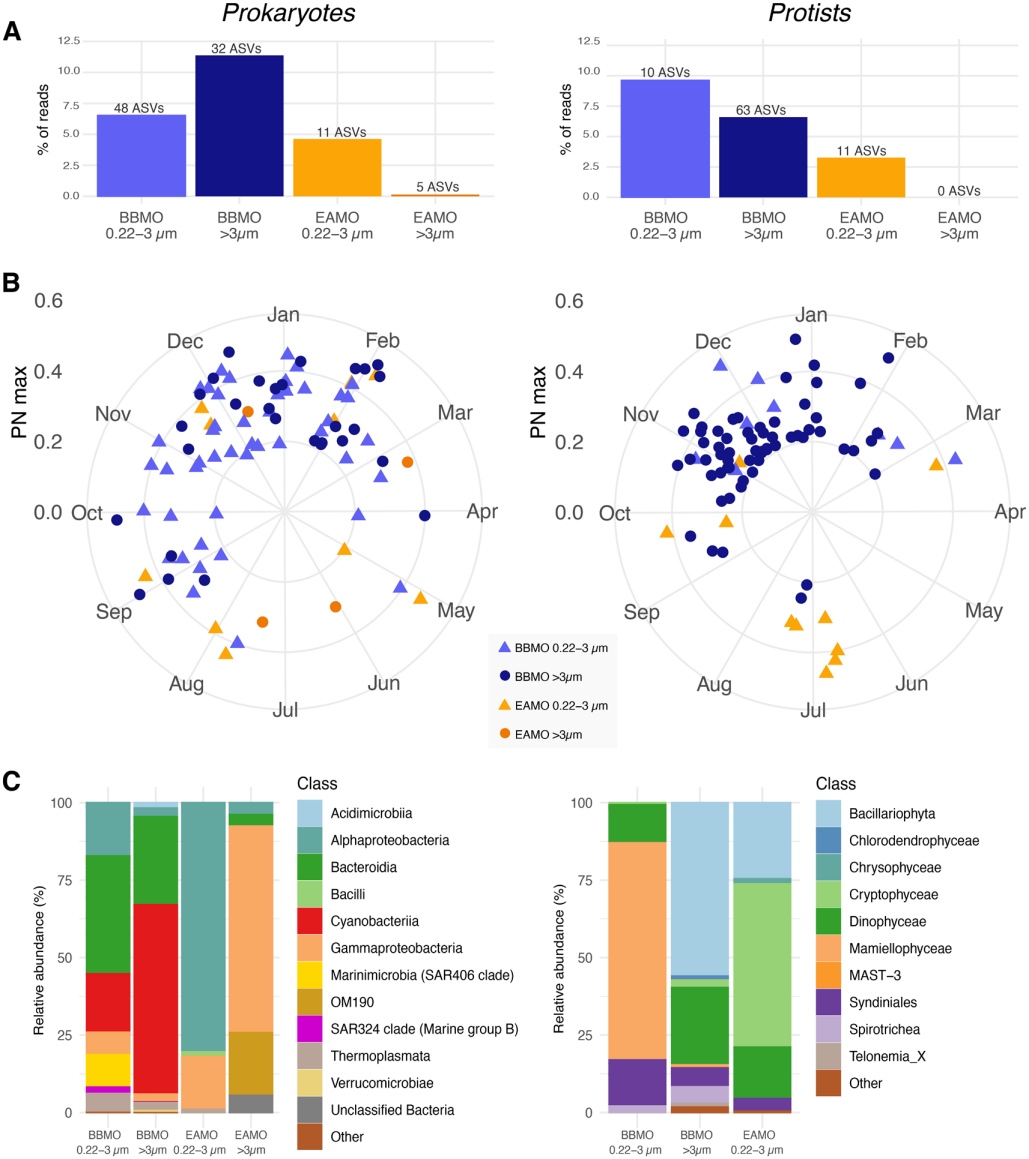
Seasonal ASVs in the temperate site (BBMO) and the tropical site (EAMO). (A) Percentage of reads and number of seasonal ASVs in each site and size‐fraction. (B) Polar plots representing the seasonal ASVs of the prokaryotic and protist communities. Higher strength recurrence values (PN max) represent stronger seasonal signals, and the polar plot indicates the month in the year when the maximal abundance occurs. Figure S16 shows the same, but with seasonal ASVs coloured by their taxonomic groups. (C) Taxonomy of the seasonal ASVs for prokaryotes (left) and protists (right) at each site and size‐fraction. Note that not a single ASV could be considered seasonal in the EAMO protist large size‐fraction. ASV, amplicon sequence variants; BBMO, Blanes Bay Microbial Observatory; EAMO, Equatorial Atlantic Microbial Observatory.

Our LSP (Lomb Scargle periodogram) analysis to detect seasonal ASVs revealed pronounced differences in their temporal dynamics between the two sites. From the total of 180 ASVs identified as seasonal across all datasets, the vast majority (85% or 153 ASVs) belonged to the temperate site BBMO, while only 27 seasonal ASVs (15%) were detected at the equatorial site EAMO (Figure 5A). The number of seasonal ASVs was comparable across domains (96 prokaryotes vs. 84 protists) and across size‐fractions, indicating that the pronounced seasonal signal in the BBMO was not limited to one group or size‐fraction. At the BBMO, seasonal prokaryotes accounted for ~9% of the total reads (6.54% in the free‐living and 11.38% in the particle‐associated fractions), while in the EAMO they represented < 3% of the reads (4.63% and 0.15%, respectively). Similarly, seasonal protists comprised 8.12% of the total reads at the BBMO (9.63% for small protists and 6.6% for large protists), compared to just 3.27% in the EAMO. Strikingly, none of the large protist ASVs at the EAMO passed the seasonality threshold in sharp contrast with the BBMO.

For each seasonal ASV, we extracted the month of the year with maximal abundance (Figure 5B). Seasonal ASVs at the BBMO displayed strong winter dominance, with their peak abundances occurring mainly between September and March—particularly in December, January and February. Most seasonal prokaryotes in the BBMO were associated with the free‐living fraction and included taxa from Alphaproteobacteria, Bacteroidia and Gammaproteobacteria (Figure 5C). Interestingly, only one *Synechococcus* CC9902 ASV (Cyanobacteria) was detected as seasonal in the free‐living fraction (peaking in April), while three others were seasonal in the particle‐associated fraction. Groups such as Thermoplasmata (6 ASVs) and SAR324 clade (3 ASVs) exhibited clear seasonal peaks in the winter months, whereas other ASVs showed more variable timing (Figure S16). For protists, most seasonal ASVs at the BBMO were in the large fraction (> 3 μm) taxa that peaked in late autumn and winter (November, December and January). These included several diatoms (Bacillariophyta), dinoflagellates (Dinophyceae) and parasitic taxa such as Syndiniales (Figure 5C). Among the few small protist seasonal ASVs, *Bathycoccus prasinos* (ASV_7, Mamiellophyceae) stood out, with winter peaks in February that accounted for up to 30% of the total protist reads in some samples.

In contrast, seasonal ASVs in the EAMO showed no clear temporal clustering (Figure 5B). Prokaryotic seasonal ASVs were scattered throughout the year, with individual ASVs peaking in different months. For example, SAR11 (ASV_8) peaked in December, Haliaceae (ASV_600) in July and a Rhodobacteraceae ASV (ASV_4640) in March. Interestingly, among the few seasonal protists in the EAMO, half of the small size fraction ASVs (6 out of 11) peaked in July (rainy period) and were primarily members of Cryptophyceae, Bacillariophyta and Dinophyceae (Figure 5C). Still, these patterns were less coherent than those observed at the BBMO, highlighting the absence of strong seasonal structuring in the equatorial community.

### 3.4 Equatorial Community Variation Is Driven More by Biological and Stochastic Processes Than by Seasonal Selection

**FIGURE 6:**
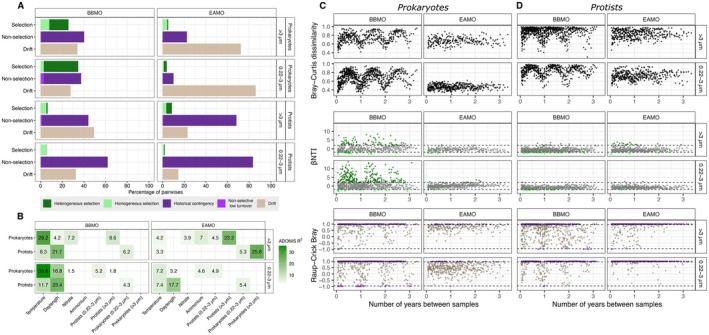
Microbial community assembly processes and environmental drivers in the contrasting time‐series. (A) Relative importance of the different ecological mechanisms structuring the microbial communities in the BBMO and the EAMO. (B) Percentage of variance (Adonis *R* ^2^) in eukaryotic and prokaryotic community composition (Bray–Curtis dissimilarity) explained by the biological and environmental variables with significant values (*p* < 0.01) in at least one of the subsets. Blank spaces depict non‐significant results (*p* > 0.01). (C, D) Time‐decay in prokaryotic and protist communities in the temperate and tropical time‐series. Bray–Curtis dissimilarity, βNTI and Raup‐Crick Bray between samples for each size‐fraction of (C) prokaryotes and (D) protists plotted against the time lag between samples. BBMO, Blanes Bay Microbial Observatory; EAMO, Equatorial Atlantic Microbial Observatory.

We observed striking differences in the relative importance of ecological processes structuring microbial communities between the two observatories (Figure 6A). Across all comparisons, deterministic selection explained a higher proportion of community turnover in prokaryotes than in protists, regardless of site or size‐fraction. Moreover, selection consistently played a stronger role in the free‐living (0.22–3 μm) fraction than in the particle‐associated (> 3 μm) one. Selection accounted for ~26% of prokaryotic turnover in the particle‐associated fraction, and ~35% in the free‐living fraction at the BBMO. Although overall selection was lower at the EAMO, the same pattern held: it explained ~5% of turnover in the particle‐associated fraction and ~4% in the free‐living fraction. For protists, selection explained a smaller fraction of the variation in both the BBMO (~6% in both size‐fractions) and the EAMO (8% in the larger and 2% in the smaller size‐fraction).

To investigate the biological and environmental factors determining the structure of the microbial communities in the contrasting latitudes, we used dissimilarity analysis (ADONIS). The temperate site free‐living prokaryotic community turnover was mainly explained by temperature (~39%), daylength (~17%) and secondarily by the small protist community structure (~5%) (Figure 6B). Conversely, in the equatorial site, the free‐living prokaryotic community turnover was weakly explained by temperature (~7%), daylength (~3%), ammonium concentration (~5%) and small protists (~5%) (Figure 6B). The particle‐associated prokaryotic community turnover was explained by temperature (~29%), nitrate (~7%), daylength (~4%) and large protists (~9%) in the temperate site while, in comparison, the tropical particle‐associated prokaryotic community was mainly explained by the large protists (~23%) and secondarily by the small protist community structure (5%), as well as environmental variables such as temperature (~4%), ammonium (7%) and nitrate (~4%) (Figure 6B). For small protists (0.22–3 μm), the main drivers at the BBMO were daylength (23%) and temperature (12%), followed by the prokaryotic community (4%). In EAMO, these values were consistently lower, with daylength explaining 17%, temperature 7% and prokaryotes 5%. For larger protists (> 3 μm), turnover at the BBMO was also largely explained by daylength (22%) and temperature (8%), whereas at EAMO, the main explanatory variable was particle‐associated prokaryotes (26%), followed by free‐living prokaryotes (5%) and only a minor role for temperature (3%), suggesting abiotic drivers had a negligible role in shaping large protist turnover in the equatorial site.

### 3.5 Equatorial Network Metrics Show No Association With Seasonal Environmental Factors

**FIGURE 7:**
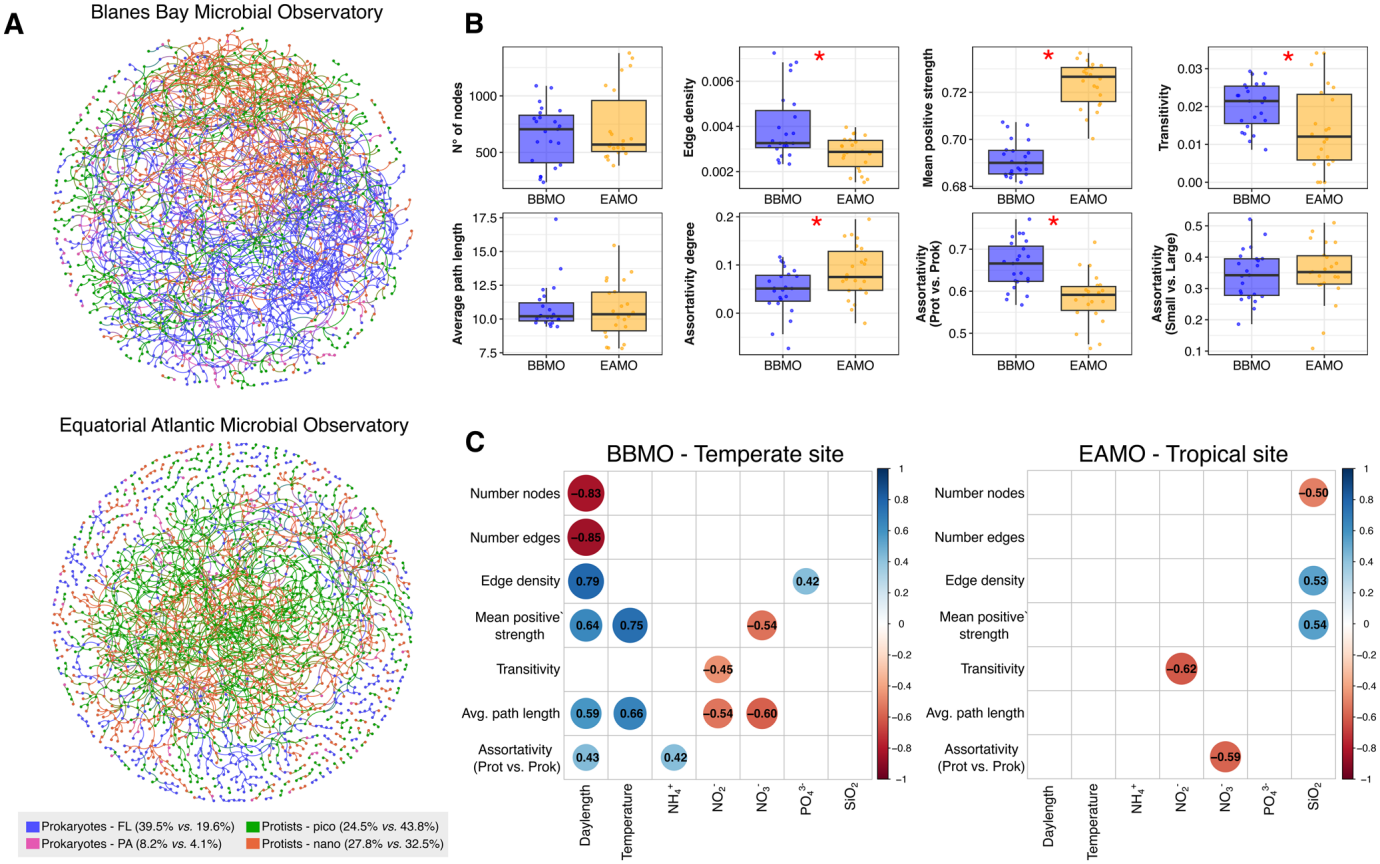
(A) Visualisation of the global static microbial networks of the BBMO and the EAMO. The percentages in the edge colour legend indicate the proportion of the biological domain (prokaryotes and protists) and size‐fraction (0.22–3 μm: Free‐living (FL) or picoplankton; and > 3 μm: Particle‐associated (PA) or nanoplankton) in each network (BBMO vs. EAMO). Figure S17 shows the static network metrics in both observatories. (B) The dynamic temporal network metrics differ between the BBMO and the EAMO. Red asterisks depict statistically significant differences (*t*‐test, *p* < 0.01). (C) Spearman correlation matrices between network metrics and environmental variables in both observatories. Empty boxes represent non‐significant correlations (*p* > 0.05). BBMO, Blanes Bay Microbial Observatory; EAMO, Equatorial Atlantic Microbial Observatory.

Microbial co‐occurrence networks revealed fundamental structural differences between the equatorial and temperate sites, both in terms of composition and topological properties (Figure 7). At EAMO, the network was strongly dominated by protists (77% vs. 24% prokaryotes), particularly in the small size fraction (Figure 7A). In contrast, BBMO showed a more balanced composition (53% vs. 48%), with the small fraction especially enriched in prokaryotes. Several network metrics also differed significantly between the two sites (Figure 7B). The temperate network exhibited higher edge density (*t* = 3.3, df = 32.09, *p* < 0.01), transitivity (*t* = 2.26, df = 32.95, *p* < 0.05) and domain‐based assortativity (*t* = 4.45, df = 42.63, *p* < 0.01), indicating greater interconnectedness and modular structure. In contrast, the equatorial network had significantly higher mean positive association strength (*t* = −12.26, df = 40.11, *p* < 0.001) and degree‐based assortativity (*t* = −2.48, df = 41.14, *p* < 0.05), suggesting that connections are concentrated among a smaller core of highly connected taxa, resulting in stronger positive associations but lower overall network complexity and modularity.

A key distinction between sites was the correlation between network structure and environmental variability (Figure 7C). At BBMO, most network metrics (except transitivity) were significantly linked to seasonal factors, especially daylength and temperature. For instance, the number of nodes and edges was negatively correlated with daylength (Spearman *r* ≈ −0.8), while edge density and mean positive association strength were positively correlated with it (*r* ≈ 0.8). In contrast, EAMO showed no significant relationships with temperature or daylength, though a few metrics correlated with nutrients, such as a positive association between edge density and silicate (*r* = 0.53) and a negative association between transitivity and nitrite (*r* = −0.62). These differences reinforce the idea that microbial community structure at low latitudes is less shaped by seasonal environmental variability.

## 4 Discussion

Our results support our central hypothesis that microbial community turnover is less strongly governed by environmental selection in the equatorial site (EAMO) than in the temperate site (BBMO), in line with the broader expectation that environmental heterogeneity drives deterministic processes. This finding is consistent with recent reports demonstrating that selection increases with environmental variability across oceanic gradients (Junger et al. 2023; Milke et al. 2022). Comparable patterns have also been observed in microbial communities from terrestrial (Dini‐Andreote et al. 2015; Stegen et al. 2013) and freshwater ecosystems (Huber et al. 2020; Vass et al. 2020). In our study, the stronger role of selection in the temperate site was supported by multiple lines of evidence: (i) a higher proportion of turnover explained by selection in the probabilistic ecological model, (ii) the greater proportion of beta‐diversity explained by environmental variables such as daylength and temperature, (iii) the higher number and relative abundance of seasonal ASVs and (iv) stronger coupling between network metrics and seasonal variables in the temperate site than in the equatorial site. Accordingly, classic studies have long noted that tropical plankton communities exhibit lower amplitude in population fluctuations, with little evidence for ecological succession or phenology (Connell and Orias 1964; Dunbar 1960), in contrast to the strong seasonal successions characteristic of temperate systems (Margalef 1978). This latitudinal gradient in environmental variability likely underpins the stronger deterministic structuring and seasonal recurrence observed in the BBMO when compared to the EAMO. Additionally, higher metabolic rates in tropical environments (Amado et al. 2013) can amplify stochasticity by increasing mutation rates, intrinsic mortality and demographic fluctuations, thereby enhancing the role of ecological drift in shaping community composition (Saito et al. 2021).

We also found marked differences between biological domains. Prokaryotic turnover was more strongly explained by selection than protist turnover across both sites and size‐fractions. This aligns with previous findings from both marine (Junger et al. 2023; Logares et al. 2020; Milke et al. 2022, 2023) and freshwater systems (Logares et al. 2018; Vass et al. 2020), where selection more consistently structures prokaryotic communities. These differences likely stem from domain‐specific traits: protists tend to be larger, with smaller population sizes, which limits their dispersal (Massana and Logares 2013; Villarino et al. 2018) and increase the potential effects of ecological drift (Bie et al. 2012; Fodelianakis et al. 2021). Prokaryotes, by contrast, exhibit wider dispersal and a higher potential for dormancy—a mechanism less common among marine protists—which may buffer them from stochastic processes and reinforce the signature of homogeneous selection (Lennon et al. 2021; Locey et al. 2020). Nonetheless, some protists can form long‐lived resistant cysts or spores, with known persistence ranging from months in the water column (Ellegaard and Ribeiro 2018) to centuries or millennia in sediments (Sanyal et al. 2022). We also observed that selection played a relatively larger role in structuring free‐living (0.22–3 μm) than particle‐associated (> 3 μm) prokaryotic communities, consistent with previous observations in the Atlantic and Pacific Oceans (Milke et al. 2022, 2023). Larger particles or hosts can provide microhabitats that buffer environmental variability (Yung et al. 2016), reducing the strength of bulk environmental selection (Mestre et al. 2020), and their spatial confinement likely imposes stronger dispersal limitation and drift on associated microbes. For instance, copepod‐associated microbial communities are more shaped by drift than environmental selection than surrounding seawater communities (Velasquez et al. 2025). Moreover, we found stronger historical contingency for protists than prokaryotes in both sites. The higher number of common prokaryotes (*n* = 48) compared to protists (*n* = 21) further supports that historical contingency, mediated by dispersal limitation, is stronger for protists. Dispersal limitation strengthens historical contingency by making communities more dependent on colonisation history and associated priority effects (Fukami 2015; Nemergut et al. 2013).

Beyond ecological processes, we also observed clear differences in diversity and community composition between observatories. Prokaryotic diversity was, on average, significantly higher for both size‐fractions at the temperate site (BBMO), particularly in winter, while protist diversity was higher only in the small size fraction at the equatorial site (EAMO). These results partially corroborate global spatial observations showing prokaryotic diversity plateauing at mid‐latitudes and microeukaryotic diversity increasing toward the tropics (Ibarbalz et al. 2019). Time‐series comparisons further indicate that the Australian temperate observatories harbour comparable or higher prokaryotic diversity than tropical sites (Raes et al. 2024), consistent with the broad optimal thermal range for prokaryotes (15°C–30°C) observed at mid‐ to low latitudes (Ibarbalz et al. 2019). One possible explanation for the higher mean prokaryotic diversity at the temperate site, which peaks in winter, is the stronger seasonal turnover characteristic of mid‐latitudes (Fuhrman et al. 2015; Ladau et al. 2013). To our knowledge, no global direct comparisons of protist diversity across observatories have been published, and our study provides the first time‐series comparison between sites at contrasting latitudes. Across both sites, protists exhibited higher temporal turnover (beta‐diversity) than prokaryotes, consistent with long‐term observations at the San‐Pedro Ocean Time‐Series (Yeh and Fuhrman 2022).

Latitudinal differences were also evident in the number of site‐indicator and seasonal ASVs. The higher number of EAMO‐indicators compared to BBMO‐indicators is likely explained by the greater stability in diversity at the equatorial site. Among the most abundant prokaryotic EAMO‐indicators, we found ASVs assigned to *Synechococcus* CC9902, SAR406, *Cyanobium* PCC‐6307 and the candidate actinobacterial genus *Actinomarina*. These bacterial genera have been previously reported in equatorial coastal waters of Singapore (Chénard et al. 2019; Wijaya et al. 2023), supporting their widespread distribution in low‐latitude coastal ecosystems. We detected far fewer seasonal ASVs in the equatorial site (*n* = 16) than in the temperate site (*n* = 80). Notably, many common prokaryotes such as *Synechococcus* CC9902 (e.g., ASV_1 and ASV_35), *Cyanobium* PCC‐6307 (ASV_27) and several Flavobacteriales (e.g., ASV_13, ASV_14, ASV_19) were seasonal in the BBMO but not in the EAMO. Conversely, only a few common taxa, such as SAR11 Clade I (ASV_8) and Clade II (ASV_10), and the SAR86 clade (Gammaproteobacteria, ASV_103), along with abundant EAMO‐indicators such as SAR116 (ASV_31) and OM182 (Gammaproteobacteria, ASV_116), displayed recurrent patterns at the EAMO. This reinforces the reduced influence of seasonal environmental selection in tropical waters.

Protist EAMO‐indicators included several green algae assigned to Mamiellophyceae (*Micromonas* clade B5, *Ostreococcus* tauri and *O*. clade B), as well as the fast‐growing, thermo‐halotolerant *Picochlorum*, all found year‐round in equatorial waters (Chénard et al. 2019). The aggregate‐forming diatom *Minutocellus polymorphus* (ASV_79), which showed recurrent temporal patterns in the EAMO, is typically associated with coastal and estuarine environments, with documented occurrences in tropical Eastern Pacific and Indian Ocean waters (Barry‐Martinet et al. 2025; Hernández‐Márquez et al. 2023). The MOCH‐5 group, detected here as an abundant and exclusive EAMO‐indicator, has also been reported as a major phototrophic component in the tropical North Atlantic around the Amazon River plume (Charvet et al. 2021). Parasitic Syndiniales (Dino‐Group‐I) Clade‐1 and Clade‐4, both previously observed in equatorial waters (Chénard et al. 2019), had ASVs emerging as EAMO‐indicators and BBMO‐indicators, consistent with segregation between Mediterranean and tropical/subtropical communities (Rizos et al. 2023). Other notable EAMO‐indicators showing recurrent patterns included *Oodinium pouchetii*, a parasitic dinoflagellate infecting appendicularians off the Brazilian coast (Gómez and Skovgaard 2015), the athecate dinoflagellate genus *Warnowia*, characterised by its sophisticated photoreceptor organelle (Cooney et al. 2023) and the common cryptophyte *Teleaulax* sp. (ASV_29). Collectively, these abundant equatorial indicators may serve as potential candidates to monitor environmental changes (e.g., ocean warming, marine heatwaves) in temperate sites (Brown et al. 2024) such as the BBMO.

Furthermore, differences in diversity and community structure may also reflect broader ecosystem characteristics such as trophic state and continental shelf extent. The EAMO is a more productive system, with chlorophyll‐a concentrations about twice those observed at the BBMO (Figure S1), where winter–spring blooms can reach ~1 mg m^−3^ (Auladell et al. 2022). Additionally, although narrower than some global shelves, the continental shelf off the northeastern Brazilian coast where the EAMO is located is broader, averaging ~30–40 km in width (Vital et al. 2010), than the immediate Blanes Bay coastal shelf, which is only ~4 km wide (Durán et al. 2013). Such geomorphological differences likely enhance benthic–pelagic coupling and nutrient inputs via coastal mixing and sediment resuspension (Bauer et al. 2013), potentially promoting irregular non‐seasonal variability in microbial communities at the EAMO. Indeed, studies in equatorial coastal waters have shown that monsoon seasons (Chénard et al. 2019), and local features like riverine input and tidal mixing (Wijaya et al. 2023) can drive environmental selection on microbial communities over very short time scales. In this study, the equatorial community structure was not explained by climatic variables (e.g., SOI and rainfall), and our monthly data suggest non‐seasonal nutrient variation in the EAMO, but capturing these dynamics more precisely would require higher temporal resolution than in our study. Future studies aiming to disentangle the mechanisms structuring microbial communities in equatorial observatories should consider weekly or even daily sampling in relation to tidal cycles, or during critical periods such as the rainy (April–July) and windy (August–November) months in the EAMO region.

The structure of microbial co‐occurrence networks further emphasised latitudinal contrasts in assembly mechanisms. The stronger dominance of protists in the EAMO network likely reflects its higher protist diversity compared to BBMO, particularly in the small size fraction, thereby increasing their prominence and inferred associations in co‐occurrence networks (Lima‐Mendez et al. 2015). The EAMO network was characterised by lower connectivity but stronger positive association strength and higher assortativity by degree, implying a community with fewer but more stable interactions, potentially maintained by biotic interactions and environmental homogeneity. The higher average path length in EAMO networks suggests greater stability through slower disturbance propagation, as fewer direct connections and longer paths reduce cascading effects in more homogeneous environments (Coyte et al. 2015; Deutschmann et al. 2024). In contrast, the BBMO network exhibited higher edge density, transitivity and domain‐based assortativity, indicating more modular and cohesive microbial interactions (Deutschmann et al. 2023). These patterns may reflect both a higher number of co‐occurring taxa and more predictable seasonal recurrences at BBMO, consistent with classic ecological theory (Margalef 1978) and microbial time‐series studies showing seasonal succession driven by light and temperature (Auladell et al. 2022; Caracciolo et al. 2022; Faust and Raes 2012; Gilbert et al. 2012; Lambert et al. 2019; Yeh and Fuhrman 2022).

The strong correlation between network metrics and seasonal variables at the BBMO, but not at the EAMO, suggests that environmental selection structures not just community composition, but also microbial interaction networks in the temperate ocean (Deutschmann et al. 2023). The EAMO network metrics, however, correlated with nitrate and silicate concentrations, reinforcing that the microbial community structure is likely being affected by non‐seasonal episodic sedimentary mixing through tidal currents (Wijaya et al. 2023) in the equatorial site. For example, the positive correlation between edge density and positive strength with silicate suggests sediment resuspension could lead to a more stable and connected microbial community in the EAMO. Overall, our network and ecological analyses converge on a coherent view: tropical microbial communities, while less connected, are more stable and dominated by stochastic or biotic processes, whereas temperate communities are shaped by stronger environmental selection that also influences the structure and dynamics of microbial interactions.

We show in this study that beyond the expected differences in microbial community patterns in coastal observatories from contrasting latitudes, the ecological processes that generate these patterns are fundamentally different. Deterministic selection plays a greater role in shaping community turnover and interaction networks in the temperate site, largely due to stronger environmental gradients such as daylength and temperature. Conversely, tropical microbial communities are more temporally stable and likely more influenced by biological interaction and stochastic processes. This study further reinforces the value of comparative time‐series across latitudes for disentangling the drivers of microbial community structure in the ocean. As climate change alters the amplitude and frequency of environmental fluctuations, understanding the balance between deterministic and stochastic forces in shaping microbiomes will be essential to better predict future ecosystem responses. This underscores the need for more continuous tropical time‐series observatories, particularly those applying molecular tools, which remain underrepresented globally compared to temperate datasets.

## Author Contributions

P.C.J., V.S.K., J.M.G. and H.S. designed the study. H.S. idealised and established the EAMO. J.M.G. idealised and established the BBMO. H.S., M.M., A.M.A. and V.S.K. performed the EAMO fieldwork. H.S., V.S.K. and P.C.J. extracted the DNA from the EAMO samples. R.L., J.M.G. and I.F. sustained the long‐term BBMO sampling. P.C.J. performed the primary data analysis and visualisation. R.P. carried out the nutrient concentration analysis. P.H. assisted with the metabarcoding data processing. C.R.G. helped with the LSP analysis, while I.M.D. and S.C. helped with the network analyses. All authors assisted with interpretation of the data. P.C.J., V.S.K. and H.S. wrote the manuscript. All authors contributed substantially to manuscript revisions.

## Funding

This work was supported by Fundação de Amparo à Pesquisa do Estado de São Paulo (FAPESP), 2014/13139‐3, 2017/26786‐1, 2020/02517‐4; Conselho Nacional de Desenvolvimento Científico e Tecnológico (CNPQ), 474759/2013‐0, 303906/2021‐9, 313784/2023‐0, PVE 400313‐2014‐6; Coordenação de Aperfeiçoamento de Pessoal de Nível Superior (CAPES), BJT 013/2012; Fundação de Apoio Institucional ao Desenvolvimento Científico e Tecnológico (FAI/UFSCar), 3213/2020‐83; Horizon 2020 Framework Programme, 862923; European Commission, FP7‐ENV‐2012‐308392; Ministerio de Ciencia, Innovación y Universidades, PID2019‐105775RB‐I00, PID2022‐136281NB‐I00, CEX2024‐001494‐S; Agencia Santafesina de Ciencia, Tecnología e Innovación, 117/14.

## Disclosure

*Benefit‐Sharing Statement*: Benefits from this research stem from the sharing of our data and results on public databases as described below.

## Conflicts of Interest

The authors declare no conflicts of interest.

## Supporting information


**Data S1:** mec70241‐sup‐0001‐Supinfo.pdf.

## Data Availability

The raw DNA sequences obtained in this study were deposited in the European Nucleotide Archive (http://www.ebi.ac.uk/ena) under accession number PRJEB48035 for the BBMO and in the NCBI (https://www.ncbi.nlm.nih.gov/) under accession number PRJNA414763 for the EAMO. The ASV tables and corresponding taxonomic classifications are deposited in Zenodo: 10.5281/zenodo.16866531 for the 16S V4V5 and 10.5281/zenodo.16866563 for the 18S V4. The contextual and environmental data are also deposited in Zenodo: 10.5281/zenodo.16861237. The processed tables and R scripts to reproduce the analyses are available in GitHub: https://github.com/pcjunger/eamo‐bbmo/.

## References

[mec70241-bib-0001] Albert, R. , H. Jeong , and A.‐L. Barabási . 2000. “Error and Attack Tolerance of Complex Networks.” Nature 406, no. 6794: 378–382. 10.1038/35019019. 10935628

[mec70241-bib-0002] Amado, A. M. , F. Meirelles‐Pereira , L. D. O. Vidal , et al. 2013. “Tropical Freshwater Ecosystems Have Lower Bacterial Growth Efficiency Than Temperate Ones.” Frontiers in Microbiology 4: 167. 10.3389/fmicb.2013.00167. 23801986 PMC3689033

[mec70241-bib-0003] Auladell, A. , A. Barberán , R. Logares , E. Garcés , J. M. Gasol , and I. Ferrera . 2022. “Seasonal Niche Differentiation Among Closely Related Marine Bacteria.” ISME Journal 16, no. 1: 1. 10.1038/s41396-021-01053-2. 34285363 PMC8692485

[mec70241-bib-0004] Barberán, A. , S. T. Bates , E. O. Casamayor , and N. Fierer . 2012. “Using Network Analysis to Explore Co‐Occurrence Patterns in Soil Microbial Communities.” ISME Journal 6, no. 2: 343–351. 10.1038/ismej.2011.119. 21900968 PMC3260507

[mec70241-bib-0005] Barry‐Martinet, R. , T. Pollet , F. Fay , et al. 2025. “Geographic Genetic Divergence in Tychoplanktonic Taxa Dominating Diatom Communities in Marine Biofilms.” Environmental DNA 7, no. 3: e70116. 10.1002/edn3.70116.

[mec70241-bib-0006] Bastian, M. , S. Heymann , and M. Jacomy . 2009. “Gephi: An Open Source Software for Exploring and Manipulating Networks.” Proceedings of the International AAAI Conference on Web and Social Media 3, no. 1: 361–362. 10.1609/icwsm.v3i1.13937.

[mec70241-bib-0007] Bauer, J. E. , W.‐J. Cai , P. A. Raymond , T. S. Bianchi , C. S. Hopkinson , and P. A. G. Regnier . 2013. “The Changing Carbon Cycle of the Coastal Ocean.” Nature 504, no. 7478: 61–70. 10.1038/nature12857. 24305149

[mec70241-bib-0008] Behrenfeld, M. J. , R. T. O'Malley , D. A. Siegel , et al. 2006. “Climate‐Driven Trends in Contemporary Ocean Productivity.” Nature 444, no. 7120: 752–755. 10.1038/nature05317. 17151666

[mec70241-bib-0009] Bie, T. , L. Meester , L. Brendonck , et al. 2012. “Body Size and Dispersal Mode as Key Traits Determining Metacommunity Structure of Aquatic Organisms.” Ecology Letters 15, no. 7: 740–747. 10.1111/j.1461-0248.2012.01794.x. 22583795

[mec70241-bib-0010] Brown, M. V. , M. Ostrowski , L. F. Messer , et al. 2024. “A Marine Heatwave Drives Significant Shifts in Pelagic Microbiology.” Communications Biology 7, no. 1: 125. 10.1038/s42003-023-05702-4. 38267685 PMC10808424

[mec70241-bib-0011] Brown, M. V. , J. van de Kamp , M. Ostrowski , et al. 2018. “Systematic, Continental Scale Temporal Monitoring of Marine Pelagic Microbiota by the Australian Marine Microbial Biodiversity Initiative.” Scientific Data 5, no. 1: 180130. 10.1038/sdata.2018.130. 30015804 PMC6049030

[mec70241-bib-0012] Buttigieg, P. L. , E. Fadeev , C. Bienhold , L. Hehemann , P. Offre , and A. Boetius . 2018. “Marine Microbes in 4D—Using Time Series Observation to Assess the Dynamics of the Ocean Microbiome and Its Links to Ocean Health.” Current Opinion in Microbiology 43: 169–185. 10.1016/j.mib.2018.01.015. 29477022

[mec70241-bib-0013] Cáceres, M. D. , and P. Legendre . 2009. “Associations Between Species and Groups of Sites: Indices and Statistical Inference.” Ecology 90, no. 12: 3566–3574. 10.1890/08-1823.1. 20120823

[mec70241-bib-0014] Callahan, B. J. , P. J. McMurdie , M. J. Rosen , A. W. Han , A. J. A. Johnson , and S. P. Holmes . 2016. “DADA2: High‐Resolution Sample Inference From Illumina Amplicon Data.” Nature Methods 13, no. 7: 7. 10.1038/nmeth.3869. 27214047 PMC4927377

[mec70241-bib-0015] Capella‐Gutiérrez, S. , J. M. Silla‐Martínez , and T. Gabaldón . 2009. “trimAl: A Tool for Automated Alignment Trimming in Large‐Scale Phylogenetic Analyses.” Bioinformatics 25, no. 15: 1972–1973. 10.1093/bioinformatics/btp348. 19505945 PMC2712344

[mec70241-bib-0016] Caracciolo, M. , F. Rigaut‐Jalabert , S. Romac , et al. 2022. “Seasonal Dynamics of Marine Protist Communities in Tidally Mixed Coastal Waters.” Molecular Ecology 31, no. 14: 3761–3783. 10.1111/mec.16539. 35593305 PMC9543310

[mec70241-bib-0017] Catlett, D. , P. G. Matson , C. A. Carlson , E. G. Wilbanks , D. A. Siegel , and M. D. Iglesias‐Rodriguez . 2020. “Evaluation of Accuracy and Precision in an Amplicon Sequencing Workflow for Marine Protist Communities.” Limnology and Oceanography: Methods 18, no. 1: 20–40. 10.1002/lom3.10343.

[mec70241-bib-0018] Chaffron, S. , E. Delage , M. Budinich , et al. 2021. “Environmental Vulnerability of the Global Ocean Epipelagic Plankton Community Interactome.” Science Advances 7, no. 35: eabg1921. 10.1126/sciadv.abg1921. 34452910 PMC8397264

[mec70241-bib-0019] Charvet, S. , E. Kim , A. Subramaniam , J. Montoya , and S. Duhamel . 2021. “Small Pigmented Eukaryote Assemblages of the Western Tropical North Atlantic Around the Amazon River Plume During Spring Discharge.” Scientific Reports 11, no. 1: 16200. 10.1038/s41598-021-95676-2. 34376772 PMC8355221

[mec70241-bib-0020] Chase, J. M. , and J. A. Myers . 2011. “Disentangling the Importance of Ecological Niches From Stochastic Processes Across Scales.” Philosophical Transactions of the Royal Society, B: Biological Sciences 366, no. 1576: 2351–2363. 10.1098/rstb.2011.0063 PMC3130433 21768151

[mec70241-bib-0021] Chénard, C. , W. Wijaya , D. Vaulot , et al. 2019. “Temporal and Spatial Dynamics of Bacteria, Archaea and Protists in Equatorial Coastal Waters.” Scientific Reports 9, no. 1: 16390. 10.1038/s41598-019-52648-x. 31704973 PMC6841670

[mec70241-bib-0022] Connell, J. H. , and E. Orias . 1964. “The Ecological Regulation of Species Diversity.” American Naturalist 98, no. 903: 399–414. 10.1086/282335.

[mec70241-bib-0023] Cooney, E. C. , C. C. Holt , V. K. L. Jacko‐Reynolds , B. S. Leander , and P. J. Keeling . 2023. “Photosystems in the Eye‐Like Organelles of Heterotrophic *Warnowiid dinoflagellates* .” Current Biology 33, no. 19: 4252–4260. 10.1016/j.cub.2023.08.052. 37703877

[mec70241-bib-0024] Coyte, K. Z. , J. Schluter , and K. R. Foster . 2015. “The Ecology of the Microbiome: Networks, Competition, and Stability.” Science 350, no. 6261: 663–666. 10.1126/science.aad2602. 26542567

[mec70241-bib-0025] Cram, J. A. , L. C. Xia , D. M. Needham , R. Sachdeva , F. Sun , and J. A. Fuhrman . 2015. “Cross‐Depth Analysis of Marine Bacterial Networks Suggests Downward Propagation of Temporal Changes.” ISME Journal 9, no. 12: 2573–2586. 10.1038/ismej.2015.76. 25989373 PMC4817623

[mec70241-bib-0026] Csardi, G. , and T. Nepusz . 2006. “The Igraph Software Package for Complex Network Research.” International Journal of Complex Systems 1695: 1–9.

[mec70241-bib-0027] de Vargas, C. , S. Audic , N. Henry , et al. 2015. “Eukaryotic Plankton Diversity in the Sunlit Ocean.” Science 348, no. 6237: 1261605. 10.1126/science.1261605. 25999516

[mec70241-bib-0028] Deutschmann, I. M. , E. Delage , C. R. Giner , et al. 2024. “Disentangling Microbial Networks Across Pelagic Zones in the Tropical and Subtropical Global Ocean.” Nature Communications 15, no. 1: 126. 10.1038/s41467-023-44550-y. PMC10762198 38168083

[mec70241-bib-0029] Deutschmann, I. M. , A. K. Krabberød , F. Latorre , et al. 2023. “Disentangling Temporal Associations in Marine Microbial Networks.” Microbiome 11, no. 1: 83. 10.1186/s40168-023-01523-z. 37081491 PMC10120119

[mec70241-bib-0030] Deutschmann, I. M. , G. Lima‐Mendez , A. K. Krabberød , et al. 2021. “Disentangling Environmental Effects in Microbial Association Networks.” Microbiome 9, no. 1: 232. 10.1186/s40168-021-01141-7. 34823593 PMC8620190

[mec70241-bib-0031] Dini‐Andreote, F. , J. C. Stegen , J. D. van Elsas , and J. F. Salles . 2015. “Disentangling Mechanisms That Mediate the Balance Between Stochastic and Deterministic Processes in Microbial Succession.” Proceedings of the National Academy of Sciences 112, no. 11: E1326–E1332. 10.1073/pnas.1414261112. PMC4371938 25733885

[mec70241-bib-0032] Doney, S. C. , M. Ruckelshaus , J. E. Duffy , et al. 2012. “Climate Change Impacts on Marine Ecosystems.” Annual Review of Marine Science 4: 11–37. 10.1146/annurev-marine-041911-111611. 22457967

[mec70241-bib-0033] Dunbar, M. J. 1960. “The Evolution of Stability in Marine Environments Natural Selection at the Level of the Ecosystem.” American Naturalist 94, no. 875: 129–136. 10.1086/282114.

[mec70241-bib-0034] Dunne, J. A. , R. J. Williams , and N. D. Martinez . 2002. “Network Structure and Biodiversity Loss in Food Webs: Robustness Increases With Connectance.” Ecology Letters 5, no. 4: 558–567. 10.1046/j.1461-0248.2002.00354.x.

[mec70241-bib-0035] Durán, R. , M. Canals , G. Lastras , et al. 2013. “Sediment Dynamics and Post‐Glacial Evolution of the Continental Shelf Around the Blanes Submarine Canyon Head (NW Mediterranean).” Progress in Oceanography 118: 28–46. 10.1016/j.pocean.2013.07.031.

[mec70241-bib-0036] Ellegaard, M. , and S. Ribeiro . 2018. “The Long‐Term Persistence of Phytoplankton Resting Stages in Aquatic ‘Seed Banks.’.” Biological Reviews 93, no. 1: 166–183. 10.1111/brv.12338. 28474820

[mec70241-bib-0037] Falkowski, P. G. , T. Fenchel , and E. F. Delong . 2008. “The Microbial Engines That Drive Earth's Biogeochemical Cycles.” Science 320, no. 5879: 1034–1039. 10.1126/science.1153213. 18497287

[mec70241-bib-0038] Faust, K. , and J. Raes . 2012. “Microbial Interactions: From Networks to Models.” Nature Reviews Microbiology 10, no. 8: 538–550. 10.1038/nrmicro2832. 22796884

[mec70241-bib-0039] Ferrera, I. , A. Auladell , V. Balagué , et al. 2024. “Seasonal and Interannual Variability of the Free‐Living and Particle‐Associated Bacteria of a Coastal Microbiome.” Environmental Microbiology Reports 16, no. 4: e13299. 10.1111/1758-2229.13299. 39081120 PMC11289420

[mec70241-bib-0040] Fodelianakis, S. , A. Valenzuela‐Cuevas , A. Barozzi , and D. Daffonchio . 2021. “Direct Quantification of Ecological Drift at the Population Level in Synthetic Bacterial Communities.” ISME Journal 15, no. 1: 1. 10.1038/s41396-020-00754-4. 32855435 PMC7852547

[mec70241-bib-0041] Fuhrman, J. A. , J. A. Cram , and D. M. Needham . 2015. “Marine Microbial Community Dynamics and Their Ecological Interpretation.” Nature Reviews Microbiology 13, no. 3: 133–146. 10.1038/nrmicro3417. 25659323

[mec70241-bib-0042] Fukami, T. 2015. “Historical Contingency in Community Assembly: Integrating Niches, Species Pools, and Priority Effects.” Annual Review of Ecology, Evolution, and Systematics 46: 1–23. 10.1146/annurev-ecolsys-110411-160340.

[mec70241-bib-0122] Gasol, J. M. , C. Cardelús , X. A. Morán , et al. 2016. “Seasonal Patterns in Phytoplankton Photosynthetic Parameters and Primary Production at a Coastal NW Mediterranean Site.” Scientia Marina 80, no. S1: 63–77. 10.3989/scimar.04480.06e.

[mec70241-bib-0043] Gasol, J. M. , and P. A. del Giorgio . 2000. “Using Flow Cytometry for Counting Natural Planktonic Bacteria and Understanding the Structure of Planktonic Bacterial Communities.” Scientia Marina 64, no. 2: 197–224. 10.3989/scimar.2000.64n2197.

[mec70241-bib-0044] Gazulla, C. R. , A. Auladell , C. Ruiz‐González , et al. 2022. “Global Diversity and Distribution of Aerobic Anoxygenic Phototrophs in the Tropical and Subtropical Oceans.” Environmental Microbiology 24, no. 5: 2222–2238. 10.1111/1462-2920.15835. 35084095

[mec70241-bib-0045] Gilbert, J. A. , J. A. Steele , J. G. Caporaso , et al. 2012. “Defining Seasonal Marine Microbial Community Dynamics.” ISME Journal 6, no. 2: 298–308. 10.1038/ismej.2011.107. 21850055 PMC3260500

[mec70241-bib-0046] Giner, C. R. , V. Balagué , A. K. Krabberød , et al. 2019. “Quantifying Long‐Term Recurrence in Planktonic Microbial Eukaryotes.” Molecular Ecology 28, no. 5: 923–935. 10.1111/mec.14929. 30411822

[mec70241-bib-0047] Giner, C. R. , M. C. Pernice , V. Balagué , et al. 2020. “Marked Changes in Diversity and Relative Activity of Picoeukaryotes With Depth in the World Ocean.” ISME Journal 14, no. 2: 2. 10.1038/s41396-019-0506-9. PMC6976695 31645670

[mec70241-bib-0048] Gloor, G. B. , J. M. Macklaim , V. Pawlowsky‐Glahn , and J. J. Egozcue . 2017. “Microbiome Datasets Are Compositional: And This Is Not Optional.” Frontiers in Microbiology 8: 2224. 10.3389/fmicb.2017.02224. 29187837 PMC5695134

[mec70241-bib-0049] Gómez, F. , and A. Skovgaard . 2015. “The Molecular Phylogeny of the Type‐Species of Oodinium Chatton, 1912 (Dinoflagellata: Oodiniaceae), a Highly Divergent Parasitic Dinoflagellate With Non‐Dinokaryotic Characters.” Systematic Parasitology 90, no. 2: 125–135. 10.1007/s11230-014-9538-8. 25655112

[mec70241-bib-0050] Gouy, M. , S. Guindon , and O. Gascuel . 2010. “SeaView Version 4: A Multiplatform Graphical User Interface for Sequence Alignment and Phylogenetic Tree Building.” Molecular Biology and Evolution 27, no. 2: 221–224. 10.1093/molbev/msp259. 19854763

[mec70241-bib-0051] Grasshoff, K. , K. Kremling , and M. Ehrhardt . 2009. Methods of Seawater Analysis. John Wiley & Sons.

[mec70241-bib-0052] Guidi, L. , S. Chaffron , L. Bittner , et al. 2016. “Plankton Networks Driving Carbon Export in the Oligotrophic Ocean.” Nature 532: 7600. 10.1038/nature16942. PMC4851848 26863193

[mec70241-bib-0053] Guillou, L. , D. Bachar , S. Audic , et al. 2013. “The Protist Ribosomal Reference Database (PR2): A Catalog of Unicellular Eukaryote Small Sub‐Unit rRNA Sequences With Curated Taxonomy.” Nucleic Acids Research 41, no. D1: D597–D604. 10.1093/nar/gks1160. 23193267 PMC3531120

[mec70241-bib-0054] Guo, B. , L. Zhang , H. Sun , et al. 2022. “Microbial Co‐Occurrence Network Topological Properties Link With Reactor Parameters and Reveal Importance of Low‐Abundance Genera.” npj Biofilms and Microbiomes 8, no. 1: 3. 10.1038/s41522-021-00263-y. 35039527 PMC8764041

[mec70241-bib-0055] Guo, R. , X. Ma , C. Zhu , et al. 2024. “Diversity Patterns and Ecological Assembly Mechanisms of Bacterial Communities in the Northeastern Indian Ocean Epipelagic Waters During the Northeast Monsoon.” Science of the Total Environment 951: 175755. 10.1016/j.scitotenv.2024.175755. 39182780

[mec70241-bib-0056] Hernández‐Márquez, S. , M. E. Zamudio‐Resendiz , M. L. Núñez‐Reséndiz , A. Escarcega‐Bata , and A. Sentíes . 2023. “Ultrastructural Characterization of *Minutocellus polymorphus* (Cymatosiraceae, Bacillariophyta) and First Record From the Eastern Pacific.” Botanica Marina 66, no. 2: 141–150. 10.1515/bot-2022-0052.

[mec70241-bib-0057] Holm, S. 1979. “A Simple Sequentially Rejective Multiple Test Procedure.” Scandinavian Journal of Statistics 6, no. 2: 65–70.

[mec70241-bib-0058] Huber, P. , S. Metz , F. Unrein , G. Mayora , H. Sarmento , and M. Devercelli . 2020. “Environmental Heterogeneity Determines the Ecological Processes That Govern Bacterial Metacommunity Assembly in a Floodplain River System.” ISME Journal 14, no. 12: 12. 10.1038/s41396-020-0723-2. 32719401 PMC7784992

[mec70241-bib-0059] Ibarbalz, F. M. , N. Henry , M. C. Brandão , et al. 2019. “Global Trends in Marine Plankton Diversity Across Kingdoms of Life.” Cell 179, no. 5: 1084–1097. 10.1016/j.cell.2019.10.008. 31730851 PMC6912166

[mec70241-bib-0060] Junger, P. C. , H. Sarmento , C. R. Giner , et al. 2023. “Global Biogeography of the Smallest Plankton Across Ocean Depths.” Science Advances 9, no. 45: eadg9763. 10.1126/sciadv.adg9763. 37939185 PMC10631730

[mec70241-bib-0061] Kindt, R. , and R. Coe . 2005. Tree Diversity Analysis: A Manual and Software for Common Statistical Methods for Ecological and Biodiversity Studies. World Agrofirestry Centre.

[mec70241-bib-0062] Kirchman, D. 1992. “Incorporation of Thymidine and Leucine in the Subarctic Pacific Application to Estimating Bacterial Production.” Marine Ecology Progress Series 82: 301–309. 10.3354/meps082301.

[mec70241-bib-0063] Krabberød, A. K. , I. M. Deutschmann , M. F. M. Bjorbækmo , et al. 2022. “Long‐Term Patterns of an Interconnected Core Marine Microbiota.” Environmental Microbiomes 17, no. 1: 22. 10.1186/s40793-022-00417-1. PMC9080219 35526063

[mec70241-bib-0064] Ladau, J. , T. J. Sharpton , M. M. Finucane , et al. 2013. “Global Marine Bacterial Diversity Peaks at High Latitudes in Winter.” ISME Journal 7, no. 9: 1669–1677. 10.1038/ismej.2013.37. 23514781 PMC3749493

[mec70241-bib-0065] Lambert, S. , M. Tragin , J.‐C. Lozano , et al. 2019. “Rhythmicity of Coastal Marine Picoeukaryotes, Bacteria and Archaea Despite Irregular Environmental Perturbations.” ISME Journal 13, no. 2: 388–401. 10.1038/s41396-018-0281-z. 30254323 PMC6331585

[mec70241-bib-0066] Lennon, J. T. , F. Den Hollander , M. Wilke‐Berenguer , and J. Blath . 2021. “Principles of Seed Banks and the Emergence of Complexity From Dormancy.” Nature Communications 12, no. 1: 1. 10.1038/s41467-021-24733-1. PMC8355185 34376641

[mec70241-bib-0067] Li, W. K. W. , D. V. Subba Rao , W. G. Harrison , et al. 1983. “Autotrophic Picoplankton in the Tropical Ocean.” Science 219, no. 4582: 292–295. 10.1126/science.219.4582.292. 17798278

[mec70241-bib-0068] Lima‐Mendez, G. , K. Faust , N. Henry , et al. 2015. “Determinants of Community Structure in the Global Plankton Interactome.” Science 348, no. 6237: 1262073. 10.1126/science.1262073. 25999517

[mec70241-bib-0069] Locey, K. J. , and J. T. Lennon . 2016. “Scaling Laws Predict Global Microbial Diversity.” Proceedings of the National Academy of Sciences 113, no. 21: 5970–5975. 10.1073/pnas.1521291113. PMC4889364 27140646

[mec70241-bib-0070] Locey, K. J. , M. E. Muscarella , M. L. Larsen , S. R. Bray , S. E. Jones , and J. T. Lennon . 2020. “Dormancy Dampens the Microbial Distance–Decay Relationship.” Philosophical Transactions of the Royal Society, B: Biological Sciences 375, no. 1798: 20190243. 10.1098/rstb.2019.0243. PMC7133528 32200741

[mec70241-bib-0071] Logares, R. , I. M. Deutschmann , P. C. Junger , et al. 2020. “Disentangling the Mechanisms Shaping the Surface Ocean Microbiota.” Microbiome 8, no. 1: 55. 10.1186/s40168-020-00827-8. 32312331 PMC7171866

[mec70241-bib-0072] Logares, R. , S. Sunagawa , G. Salazar , et al. 2014. “Metagenomic 16S rDNA Illumina Tags Are a Powerful Alternative to Amplicon Sequencing to Explore Diversity and Structure of Microbial Communities.” Environmental Microbiology 16, no. 9: 2659–2671. 10.1111/1462-2920.12250. 24102695

[mec70241-bib-0073] Logares, R. , S. V. M. Tesson , B. Canbäck , M. Pontarp , K. Hedlund , and K. Rengefors . 2018. “Contrasting Prevalence of Selection and Drift in the Community Structuring of Bacteria and Microbial Eukaryotes.” Environmental Microbiology 20, no. 6: 2231–2240. 10.1111/1462-2920.14265. 29727053

[mec70241-bib-0074] Lopez‐Bustins, J. A. , L. Arbiol‐Roca , J. Martin‐Vide , A. Barrera‐Escoda , and M. Prohom . 2020. “Intra‐Annual Variability of the Western Mediterranean Oscillation (WeMO) and Occurrence of Extreme Torrential Precipitation in Catalonia (NE Iberia).” Natural Hazards and Earth System Sciences 20, no. 9: 2483–2501. 10.5194/nhess-20-2483-2020.

[mec70241-bib-0075] Margalef, R. 1978. “Life‐Forms of Phytoplankton as Survival Alternatives in an Unstable Environment.” Oceanologica Acta 1, no. 4: 493–509.

[mec70241-bib-0076] Marinchel, N. , A. Marchesini , D. Nardi , et al. 2023. “Mock Community Experiments Can Inform on the Reliability of eDNA Metabarcoding Data: A Case Study on Marine Phytoplankton.” Scientific Reports 13, no. 1: 20164. 10.1038/s41598-023-47462-5. 37978238 PMC10656442

[mec70241-bib-0077] Massana, R. , and R. Logares . 2013. “Eukaryotic Versus Prokaryotic Marine Picoplankton Ecology: Marine Ecology of Picoeukaryotes and Prokaryotes.” Environmental Microbiology 15, no. 5: 1254–1261. 10.1111/1462-2920.12043. 23206217

[mec70241-bib-0078] McArdle, B. H. , and M. J. Anderson . 2001. “Fitting Multivariate Models to Community Data: A Comment on Distance‐Based Redundancy Analysis.” Ecology 82, no. 1: 290–297.

[mec70241-bib-0079] McNichol, J. , N. L. R. Williams , Y. Raut , et al. 2025. “Characterizing Organisms From Three Domains of Life With Universal Primers From Throughout the Global Ocean.” Scientific Data 12, no. 1: 1078. 10.1038/s41597-025-05423-9. 40593763 PMC12218243

[mec70241-bib-0080] Menezes, M. , P. C. Junger , V. S. Kavagutti , et al. 2023. “Temporal Patterns of Picoplankton Abundance and Metabolism on the Western Coast of the Equatorial Atlantic Ocean.” Ocean and Coastal Research 71, no. suppl 2. 10.1590/2675-2824071.22048mm.

[mec70241-bib-0081] Mestre, M. , J. Höfer , M. M. Sala , and J. M. Gasol . 2020. “Seasonal Variation of Bacterial Diversity Along the Marine Particulate Matter Continuum.” Frontiers in Microbiology 11: 1590. 10.3389/fmicb.2020.01590. 32793139 PMC7385255

[mec70241-bib-0082] Milke, F. , J. Meyerjürgens , and M. Simon . 2023. “Ecological Mechanisms and Current Systems Shape the Modular Structure of the Global Oceans' Prokaryotic Seascape.” Nature Communications 14, no. 1: 6141. 10.1038/s41467-023-41909-z. PMC10545751 37783696

[mec70241-bib-0083] Milke, F. , I. Wagner‐Doebler , G. Wienhausen , and M. Simon . 2022. “Selection, Drift and Community Interactions Shape Microbial Biogeographic Patterns in the Pacific Ocean.” ISME Journal 16, no. 12: 12. 10.1038/s41396-022-01318-4. PMC9666467 36115923

[mec70241-bib-0084] Moore, C. , J. Grewar , and G. S. Cumming . 2016. “Quantifying Network Resilience: Comparison Before and After a Major Perturbation Shows Strengths and Limitations of Network Metrics.” Journal of Applied Ecology 53, no. 3: 636–645. 10.1111/1365-2664.12486.

[mec70241-bib-0085] Moreira, D. , and P. López‐García . 2019. “Time Series Are Critical to Understand Microbial Plankton Diversity and Ecology.” Molecular Ecology 28, no. 5: 920–922. 10.1111/mec.15015. 30938044 PMC6697531

[mec70241-bib-0086] Nemergut, D. R. , S. K. Schmidt , T. Fukami , et al. 2013. “Patterns and Processes of Microbial Community Assembly.” Microbiology and Molecular Biology Reviews 77, no. 3: 342–356. 10.1128/mmbr.00051-12. 24006468 PMC3811611

[mec70241-bib-0087] Newman, M. E. J. 2002. “Assortative Mixing in Networks.” Physical Review Letters 89, no. 20: 208701. 10.1103/PhysRevLett.89.208701. 12443515

[mec70241-bib-0088] Oksanen, J. , G. L. Simpson , F. G. Blanchet , et al. 2024. “*vegan*: Community Ecology Package (Version 2.6‐6.1) (Computer Software).” The R Foundation. https://CRAN.R‐project.org/package=vegan.

[mec70241-bib-0089] Parada, A. E. , D. M. Needham , and J. A. Fuhrman . 2016. “Every Base Matters: Assessing Small Subunit rRNA Primers for Marine Microbiomes With Mock Communities, Time Series and Global Field Samples: Primers for Marine Microbiome Studies.” Environmental Microbiology 18, no. 5: 1403–1414. 10.1111/1462-2920.13023. 26271760

[mec70241-bib-0090] Platt, T. , D. V. S. Rao , and B. Irwin . 1983. “Photosynthesis of Picoplankton in the Oligotrophic Ocean.” Nature 301, no. 5902: 702–704. 10.1038/301702a0.

[mec70241-bib-0091] Price, M. N. , P. S. Dehal , and A. P. Arkin . 2009. “FastTree: Computing Large Minimum Evolution Trees With Profiles Instead of a Distance Matrix.” Molecular Biology and Evolution 26, no. 7: 1641–1650. 10.1093/molbev/msp077. 19377059 PMC2693737

[mec70241-bib-0092] Quast, C. , E. Pruesse , P. Yilmaz , et al. 2013. “The SILVA Ribosomal RNA Gene Database Project: Improved Data Processing and Web‐Based Tools.” Nucleic Acids Research 41, no. D1: D590–D596. 10.1093/nar/gks1219. 23193283 PMC3531112

[mec70241-bib-0093] R Core Team . 2013. “R: A Language and Environment for Statistical Computing.”

[mec70241-bib-0094] Raes, E. J. , S. Myles , L. MacNeil , et al. 2024. “Seasonal Patterns of Microbial Diversity Across the World Oceans.” Limnology and Oceanography Letters 9, no. 5: 512–523. 10.1002/lol2.10422.

[mec70241-bib-0095] Revelle, W. 2025. “psych: Procedures for Psychological, Psychometric, and Personality Research (Version 2.5.6) (Computer Software).” The R Foundation. https://CRAN.R‐project.org/package=psych.

[mec70241-bib-0096] Rizos, I. , P. Debeljak , T. Finet , et al. 2023. “Beyond the Limits of the Unassigned Protist Microbiome: Inferring Large‐Scale Spatio‐Temporal Patterns of Syndiniales Marine Parasites.” ISME Communications 3, no. 1: 16. 10.1038/s43705-022-00203-7. 36854980 PMC9975217

[mec70241-bib-0097] Röttjers, L. , and K. Faust . 2018. “From Hairballs to Hypotheses–Biological Insights From Microbial Networks.” FEMS Microbiology Reviews 42, no. 6: 761–780. 10.1093/femsre/fuy030. 30085090 PMC6199531

[mec70241-bib-0098] Ruf, T. 1999. “The Lomb‐Scargle Periodogram in Biological Rhythm Research: Analysis of Incomplete and Unequally Spaced Time‐Series.” Biological Rhythm Research 30, no. 2: 178–201. 10.1076/brhm.30.2.178.1422. 11708361

[mec70241-bib-0099] Saito, V. S. , D. M. Perkins , and P. Kratina . 2021. “A Metabolic Perspective of Stochastic Community Assembly.” Trends in Ecology & Evolution 36, no. 4: 280–283. 10.1016/j.tree.2021.01.003. 33536149

[mec70241-bib-0100] Salazar, G. , F. M. Cornejo‐Castillo , V. Benítez‐Barrios , et al. 2016. “Global Diversity and Biogeography of Deep‐Sea Pelagic Prokaryotes.” ISME Journal 10, no. 3: 3. 10.1038/ismej.2015.137. PMC4817678 26251871

[mec70241-bib-0101] Sanyal, A. , J. Larsson , F. Van Wirdum , et al. 2022. “Not Dead Yet: Diatom Resting Spores Can Survive in Nature for Several Millennia.” American Journal of Botany 109, no. 1: 67–82. 10.1002/ajb2.1780. 34648178

[mec70241-bib-0102] Sarmento, H. , P. Huber , C. D. Santos‐Júnior , et al. 2025. “The Southern Gap in Ocean Microbiome Science.” Ocean Microbiology 1, no. 1: 6. 10.1186/s44375-025-00006-w.

[mec70241-bib-0103] Schloss, P. D. , S. L. Westcott , T. Ryabin , et al. 2009. “Introducing Mothur: Open‐Source, Platform‐Independent, Community‐Supported Software for Describing and Comparing Microbial Communities.” Applied and Environmental Microbiology 75, no. 23: 7537–7541. 10.1128/AEM.01541-09. 19801464 PMC2786419

[mec70241-bib-0104] Solé, R. V. , and M. Montoya . 2001. “Complexity and Fragility in Ecological Networks.” Proceedings of the Royal Society of London, Series B: Biological Sciences 268, no. 1480: 2039–2045. 10.1098/rspb.2001.1767. PMC1088846 11571051

[mec70241-bib-0105] Stegen, J. C. , X. Lin , J. K. Fredrickson , et al. 2013. “Quantifying Community Assembly Processes and Identifying Features That Impose Them.” ISME Journal 7, no. 11: 93. 10.1038/ismej.2013.93. PMC3806266 23739053

[mec70241-bib-0106] Stoeck, T. , D. Bass , M. Nebel , et al. 2010. “Multiple Marker Parallel Tag Environmental DNA Sequencing Reveals a Highly Complex Eukaryotic Community in Marine Anoxic Water.” Molecular Ecology 19: 21–31. 10.1111/j.1365-294X.2009.04480.x. 20331767

[mec70241-bib-0107] Sunagawa, S. , L. P. Coelho , S. Chaffron , et al. 2015. “Structure and Function of the Global Ocean Microbiome.” Science 348, no. 6237: 1261359. 10.1126/science.1261359. 25999513

[mec70241-bib-0108] Tackmann, J. , J. F. Matias Rodrigues , and C. Von Mering . 2019. “Rapid Inference of Direct Interactions in Large‐Scale Ecological Networks From Heterogeneous Microbial Sequencing Data.” Cell Systems 9, no. 3: 286–296. 10.1016/j.cels.2019.08.002. 31542415

[mec70241-bib-0109] Tarran, G. A. , J. L. Heywood , and M. V. Zubkov . 2006. “Latitudinal Changes in the Standing Stocks of Nano‐ and Picoeukaryotic Phytoplankton in the Atlantic Ocean.” Deep Sea Research Part II: Topical Studies in Oceanography 53, no. 14: 1516–1529. 10.1016/j.dsr2.2006.05.004.

[mec70241-bib-0110] Vass, M. , A. J. Székely , E. S. Lindström , and S. Langenheder . 2020. “Using Null Models to Compare Bacterial and Microeukaryotic Metacommunity Assembly Under Shifting Environmental Conditions.” Scientific Reports 10, no. 1: 1. 10.1038/s41598-020-59182-1. 32051469 PMC7016149

[mec70241-bib-0111] Velasquez, X. , T. Ozer , M. G. Mazzocchi , et al. 2025. “Copepod‐Associated Microbial Biogeography in the Epipelagic Ocean.” Limnology and Oceanography Letters 10: lol2.70054. 10.1002/lol2.70054.

[mec70241-bib-0112] Vellend, M. 2016. The Theory of Ecological Communities. Princeton University Press; JSTOR. http://www.jstor.org/stable/j.ctt1kt82jg.

[mec70241-bib-0113] Villarino, E. , J. R. Watson , B. Jönsson , et al. 2018. “Large‐Scale Ocean Connectivity and Planktonic Body Size.” Nature Communications 9, no. 1: 1. 10.1038/s41467-017-02535-8. PMC5762663 29321528

[mec70241-bib-0114] Vital, H. , M. P. Gomes , W. F. Tabosa , E. P. Frazão , C. L. A. Santos , and J. S. P. Júnior . 2010. “Characterization of the Brazilian Continental Shelf Adjacent to Rio Grande do Norte State, NE Brazil.” Brazilian Journal of Oceanography 58: 5. 10.1590/S1679-87592010000500005.

[mec70241-bib-0115] Wang, Q. , G. M. Garrity , J. M. Tiedje , and J. R. Cole . 2007. “Naïve Bayesian Classifier for Rapid Assignment of rRNA Sequences Into the New Bacterial Taxonomy.” Applied and Environmental Microbiology 73, no. 16: 5261–5267. 10.1128/AEM.00062-07. 17586664 PMC1950982

[mec70241-bib-0116] Wang, X. , J. Zeng , F. Chen , et al. 2024. “Aridity Shapes Distinct Biogeographic and Assembly Patterns of Forest Soil Bacterial and Fungal Communities at the Regional Scale.” Science of the Total Environment 948: 174812. 10.1016/j.scitotenv.2024.174812. 39019268

[mec70241-bib-0117] Wijaya, W. , Z. Suhaimi , C. X. Chua , et al. 2023. “Frequent Pulse Disturbances Shape Resistance and Resilience in Tropical Marine Microbial Communities.” ISME Communications 3, no. 1: 55. 10.1038/s43705-023-00260-6. 37280348 PMC10244338

[mec70241-bib-0118] Yeh, Y.‐C. , and J. A. Fuhrman . 2022. “Contrasting Diversity Patterns of Prokaryotes and Protists Over Time and Depth at the San‐Pedro Ocean Time Series.” ISME Communications 2, no. 1: 36. 10.1038/s43705-022-00121-8. 37938286 PMC9723720

[mec70241-bib-0119] Yentsch, C. S. , and D. W. Menzel . 1963. “A Method for the Determination of Phytoplankton Chlorophyll and Phaeophytin by Fluorescence.” Deep Sea Research and Oceanographic Abstracts 10, no. 3: 221–231. 10.1016/0011-7471(63)90358-9.

[mec70241-bib-0120] Yung, C.‐M. , C. S. Ward , K. M. Davis , Z. I. Johnson , and D. E. Hunt . 2016. “Insensitivity of Diverse and Temporally Variable Particle‐Associated Microbial Communities to Bulk Seawater Environmental Parameters.” Applied and Environmental Microbiology 82, no. 11: 3431–3437. 10.1128/AEM.00395-16. 27037125 PMC4959251

