# Supplementary material for: Ecological Processes Shaping Marine Microbial Assemblages Diverge Between Equatorial and Temperate Time‐Series
Source: Mol Ecol. 2026 Jan 16;35(2):e70241. doi: 10.1111/mec.70241 (PMC12809624; doi:10.1111/mec.70241)
Supplement: Supplementary file 1 — Data S1: mec70241‐sup‐0001‐Supinfo.pdf. [file MEC-35-e70241-s001.pdf]

MOLECULAR ECOLOGY

Supporting Information for:

Ecological processes shaping marine microbial assemblages diverge between equatorial and temperate time-series

Pedro C. Junger, Vinicius S. Kavagutti, Ina Maria Deutschmann, Carlota R. Gazulla, Paula Huber, Maiara Menezes, Rodolfo Paranhos, André M. Amado, Isabel Ferrera, Janaina Rigonato, Samuel Chaffron, Josep M. Gasol, Ramiro Logares, Hugo Sarmento

Table of Contents:

**Material and Methods**	Pages 1-5
**Supplementary Figures**	Pages 5-18
**References**	Pages 18-19

Supporting Information

Material and methods

Analytical methods

Bacterial production (BP) rates were estimated using the [3H]-leucine incorporation method (Kirchman, 1992). For this, 15 µl of [3H]-leucine (final concentration 20 nM) were added to six 1.2 ml replicates—comprising four treatments and two dead controls (to which leucine and TCA were added prior to sample addition). The samples were incubated in the dark at in situ temperature for approximately 2.5 hours. The reaction was then halted by adding 90 µl of 100% trichloroacetic acid (TCA), after which the samples were frozen at -80°C for later analysis. Bacterial proteins were extracted by washing the samples with 5% TCA (Smith & Azam, 1992), and then measured using a Beckman LS-6500 liquid scintillation counter. Disintegration rates were converted to µg C l^{-1} h^{-1} using the conversion factor of 0.86 (Smith & Azam, 1992).

Mock community preparation for Illumina internal control

The prokaryotic mock community was prepared using near full-length amplified 16S rRNA gene clones obtained from BBMO bacterial (Alonso-Sáez et al., 2007) and archaeal (Massana et al., 2000) clone libraries. Bacterial clones were selected from different seasonal samples (Spring, Summer, and Fall; Winter samples were excluded due to unavailability of clone plates) amplified with primers 27F/1492R, ensuring broad diversity, while archaeal clones representing Euryarchaeota and Thaumarchaeota were amplified with primers 21F/958R. Selected clones were amplified using primers M13F/M13R directly from glycerol stocks (bacteria) or plasmid DNA (archaea), and the resulting PCR products were sequenced to confirm taxonomic identity and sequence quality. Clones with confirmed identity were cultured in LB medium, and plasmid DNA was extracted using a MiniPrep kit, followed by quantification using a Nanodrop spectrophotometer. The extracted plasmids were amplified with primers M13F/M13R, and the amplicons were verified by agarose gel electrophoresis to confirm expected amplicon sizes (bacterial amplicons >> archaeal amplicons). PCR products were purified using a Qiagen purification kit, quantified using a Qubit fluorometer, and tested for compatibility with sequencing primers (Parada et al., 2016) through a secondary PCR. Finally, the mock community was prepared by normalizing M13F/M13R PCR products to 20 ng/µL and combining them at a final concentration of 10 ng/µL, consistent with the sequencing facility requirements. The final set of clones included in the mock community is listed in Table S1.

Table S1. Final set of clones included in the mock community.

Clone ID	Taxonomy
AUT38	Actinobacteria - Actinomarina
AUT76	Actinobacteria - Uncultured
AUT4	Alphaproteobacteria - Erythrobacter
SPR32	Alphaproteobacteria - Rhodobacterales
SPR20	Alphaproteobacteria - SAR11 - Clade I
SUM5	Alphaproteobacteria - SAR11 - Clade I
SUM16	Alphaproteobacteria - SAR11 - Clade II

SUM19	Alphaproteobacteria - SAR11 - Clade II
SUM1	Alphaproteobacteria - SAR11 - Clade III
SUM94	Alphaproteobacteria - SAR116
AUT18	Alphaproteobacteria - Uncultured
AUT45	Alphaproteobacteria - Uncultured
AUT80	Alphaproteobacteria - Uncultured
SUM18	Bacteroidetes - NS5_marine_group
SPR33	Bacteroidetes - NS7_marine_group
AUT2	Cyanobacterium - Prochlorococcus
AUT89	Cyanobacterium - Prochlorococcus MIT0801
SUM10	Cyanobacterium - Synechococcus
AR87	Euryarchaeota
AUT3	Alphaproteobacteria - SAR11_clade
AUT16	Gammaproteobacteria - SAR86_clade
AUT27	Gammaproteobacteria - SAR86_clade
AUT55	Gammaproteobacteria - SAR86_clade
SUM93	Gammaproteobacteria - SAR86_clade
ARP3	Thaumarchaeota
SUM55	Verrucomicrobia
AUT17	Verrucomicrobia - Pelagicoccus

The prokaryotic mock community was used as a qualitative positive control to assess amplification and sequencing performance of the 16S rRNA gene V4–V5 dataset. Taxa included in the mock community were consistently recovered in the sequencing results (Figure S1), confirming primer compatibility and sequencing reliability.

A mock community was not included for the 18S rRNA gene dataset because constructing representative protist mock communities remains technically challenging and typically requires extensive cloning of long amplicons or culturing of selected taxa, often tailored to specific sites or taxonomic groups (e.g., Catlett et al., 2020; Lampe et al., 2025; Marinchel et al., 2023). Given the broad taxonomic scope of the protist community analyzed here, such an approach was beyond the scope of this study.

DNA sequencing

The DNA PCR amplification and sequencing were conducted at the Integrated Microbiome Resource (IMR, Dalhousie University, Halifax, Canada; http://imr.bio/index.html) for the 16S rRNA, and at the Functional Genomics Centre (ESALQ-USP, University of São Paulo, Piracicaba-SP, Brazil; https://sites.usp.br/cgf/) for the 18S rRNA. The 16S rRNA PCR products were normalized and purified with the Charm Biotech Just-a-Plate Purification and Normalization kit, following the sequencing facility protocol (Comeau & Kwawukume, 2023).

Determining seasonal ASVs

Function *randlps()* computes the Lomb-Scargle periodogram and the p-values for the largest peak in the periodogram by randomising the time-series sequence (Ruf, 1999). The choice of the type of normalization — “standard” or “press” — determines the values of the periodogram peaks, which are confined to the interval 0-1 if normalization = “standard”, or normalized using the factor 1/(2 * var(y)) if normalization = “press”. Studies using

the LPS approach with normalization = “standard” set the threshold at PNmax > 0.1 (Jing et al., 2024; Zhao et al., 2023), and studies with normalization = “press” set it at PNmax >10 (Auladell et al., 2022; Ferrera et al., 2024; Lambert et al., 2019).

We decided to use the default parameters in the *randlps()* function with normalization = “standard”. We manually plotted the rarefied abundance of all ASVs with PNmax > 0.1 and $p < 0.01$ across months and years, to inspect the seasonality trend (see example below with *Bathycoccus prasinos* 0.22-3 µm with PNmax = 0.35, $p < 0.001$) and decided to set the threshold to 0.2 to keep only robust signals of seasonality. Finally, since the function looks for all possible rhythmic patterns in a signal, regardless of their period, we also checked that all selected ASVs showed a period of ~1 year.

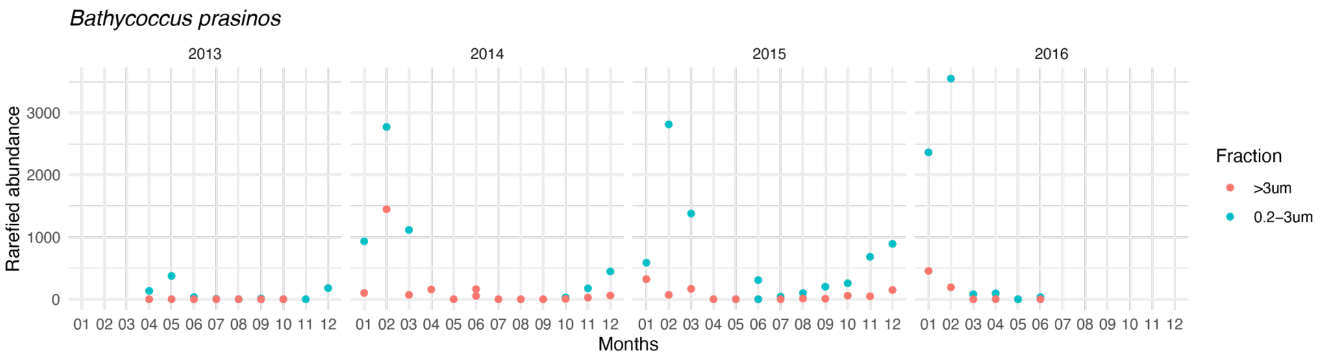

Network construction and filtering

Table S2. Summary of the sample and ASV filtering for network construction

Location		**BBMO**				**EAMO**			
Kingdom		**16S**		**18S**		**16S**		**18S**	
Size fraction		**0.22-3**	**>3**	**0.22-3**	**>3**	**0.22-3**	**>3**	**0.22-3**	**>3**
**Sample Filtering**<br>*Samples need to contain both kingdoms (16S and 18S) and both size fractions (0.22-3 µm and >3 µm)*									
#Samples		40	30	33	39	30	22	29	29
Both size-fractions within kingdom		29		31		22		27	
Both size fractions and		23				22			

both kingdoms									
**#ASV filtering done for each table (before Sample Filtering)**<br>*ASVs need to have an abundance sum above 100 counts and be present in more than 15% of samples*									
Abundance		1137	578	2547	2395	831	308	2358	2024
Prevalence		981	617	662	619	924	507	1657	1220
Both filters		818	442	628	598	655	257	1447	1083
**#ASV filtering done for samples containing both size fractions and both kingdoms**									
Both filters		797	414	577	690	578	257	1296	1114
**Size Fraction Filtering**<br>*Size fraction filtering: for each ASV, if the ratio of counts (`big size / small size`) is less than 0.5, the ASV is removed from the big size fraction. Similarly, if the ratio is greater than 2, the ASV is removed from the small size fraction.*									
#ASV removed		19	239	43	146	14	117	208	339
#ASV remaining		778	175	534	544	564	140	1088	775

Table S3. Summary of the network filtering with EnDED

Network		**BBMO**				**EAMO**			
Kingdom		**16S**		**18S**		**16S**		**18S**	
Size fraction		**0.22-3**	**3-200**	**0.22-3**	**3-200**	**0.22-3**	**3-200**	**0.22-3**	**3-200**
#ASV		778	175	534	544	564	140	1088	775
#ASV (all)		2031				2567			
**Network constructed with FlashWeave**<br>*Only nodes with at least one edge are considered, i.e., isolated nodes are removed.*									
#nodes		756	159	468	531	413	87	920	679
#nodes (all)		1914				2099			
#edges		2660 (2395 pos, 265 neg)				2435 (2073 pos, 362 neg)			
**EnDED**									

#edges removed		6 (2 pos, 4 neg)<br>daylength: 0<br>Temperature: 0<br>Salinity: 0<br>NH4: 0<br>NO2: 3<br>NO3: 1<br>PO4: 1<br>Si: 1	14 (5 pos, 9 neg)<br>daylength: 0<br>Temperature: 0<br>Salinity: 0<br>NH4: 4<br>NO2: 2<br>NO3: 2<br>PO4: 1<br>Si: 6
#edges		2654 (2393 pos, 261 neg)	2421 (2068 pos, 353 neg)
#nodes		1913	2091
**EnDED: filtering based on percentage co occurrence > 50%**			
#edges removed		40 (0 pos, 40 neg)	32 (0 pos,32 neg)
#edges		2614 (2393 pos, 221 neg)	2389 (2068 pos, 321 neg)
#nodes		1906	2074

Supplementary Figures

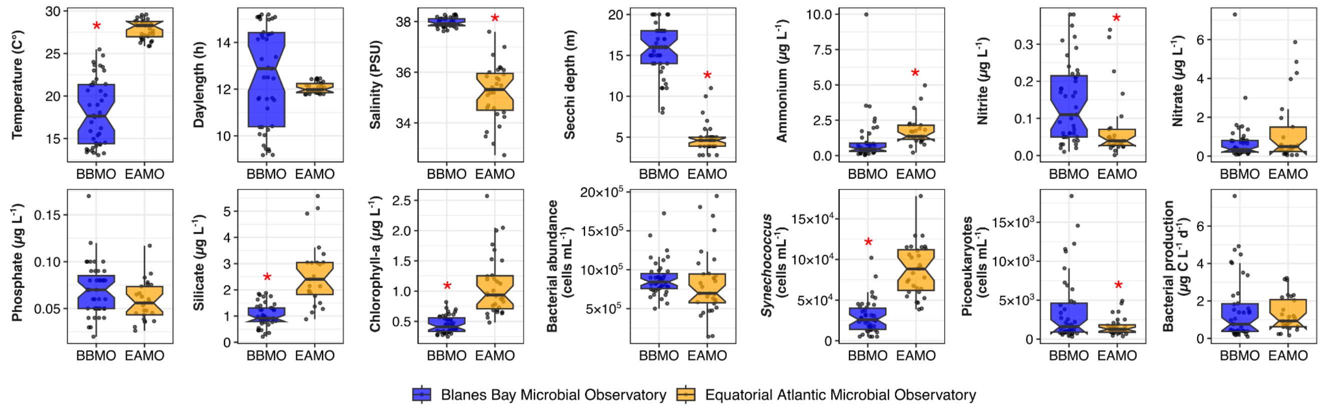

Figure S1. Comparison of environmental and biological variables between the two observatories (BBMO – Temperate site; EAMO – Tropical site). Red asterisks indicate significant statistical differences (t-test, p<0.01).

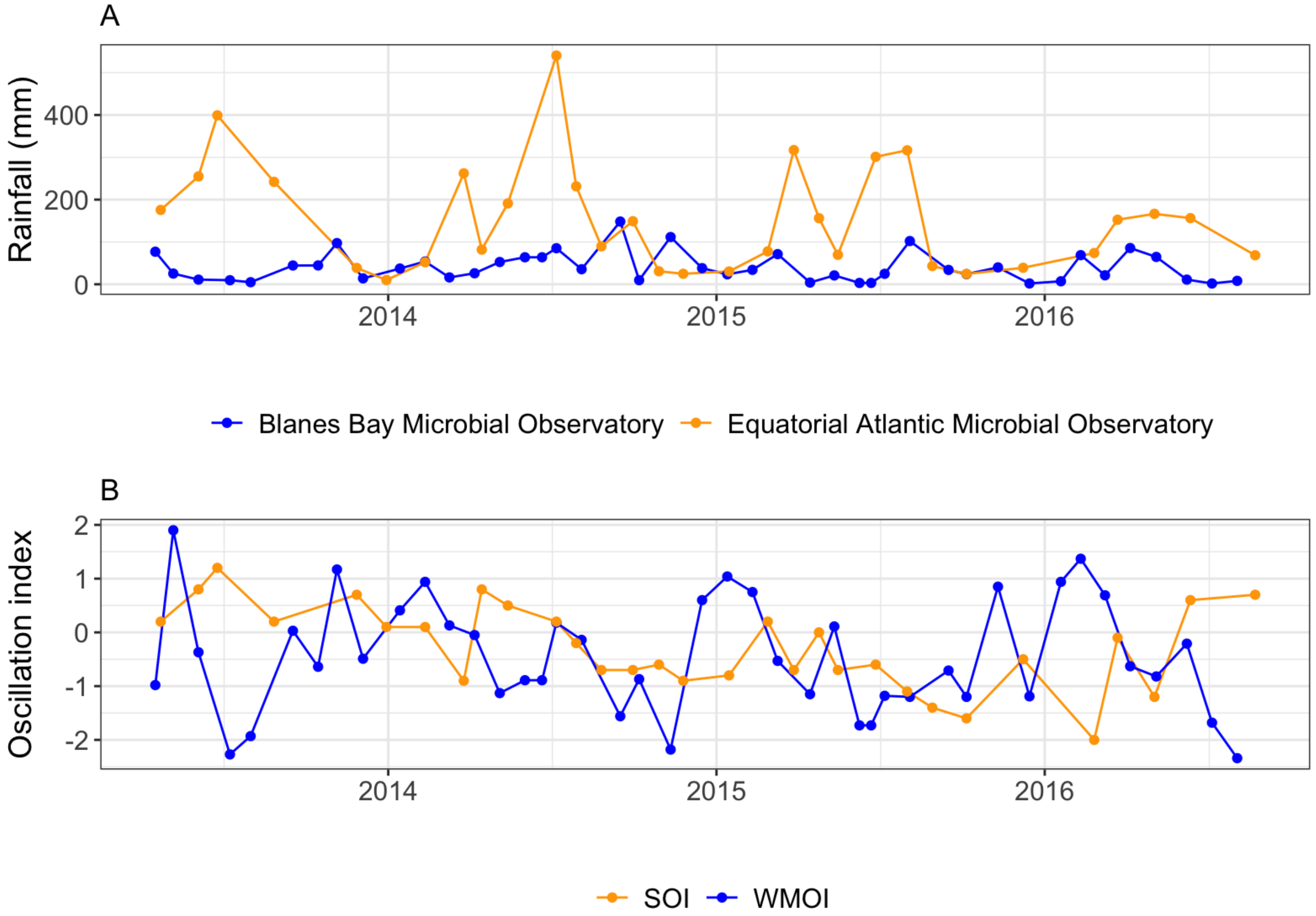

Figure S2. Time-series (April 2013 to August 2016) of the additional climatic and meteorological variables obtained from public databases, as described in the methods sections. **(A)** Monthly accumulated rainfall prior to the sampling date; **(B)** Monthly values of the Southern Oscillation (SOI) and the Western Mediterranean Oscillation (WMOI) indexes.

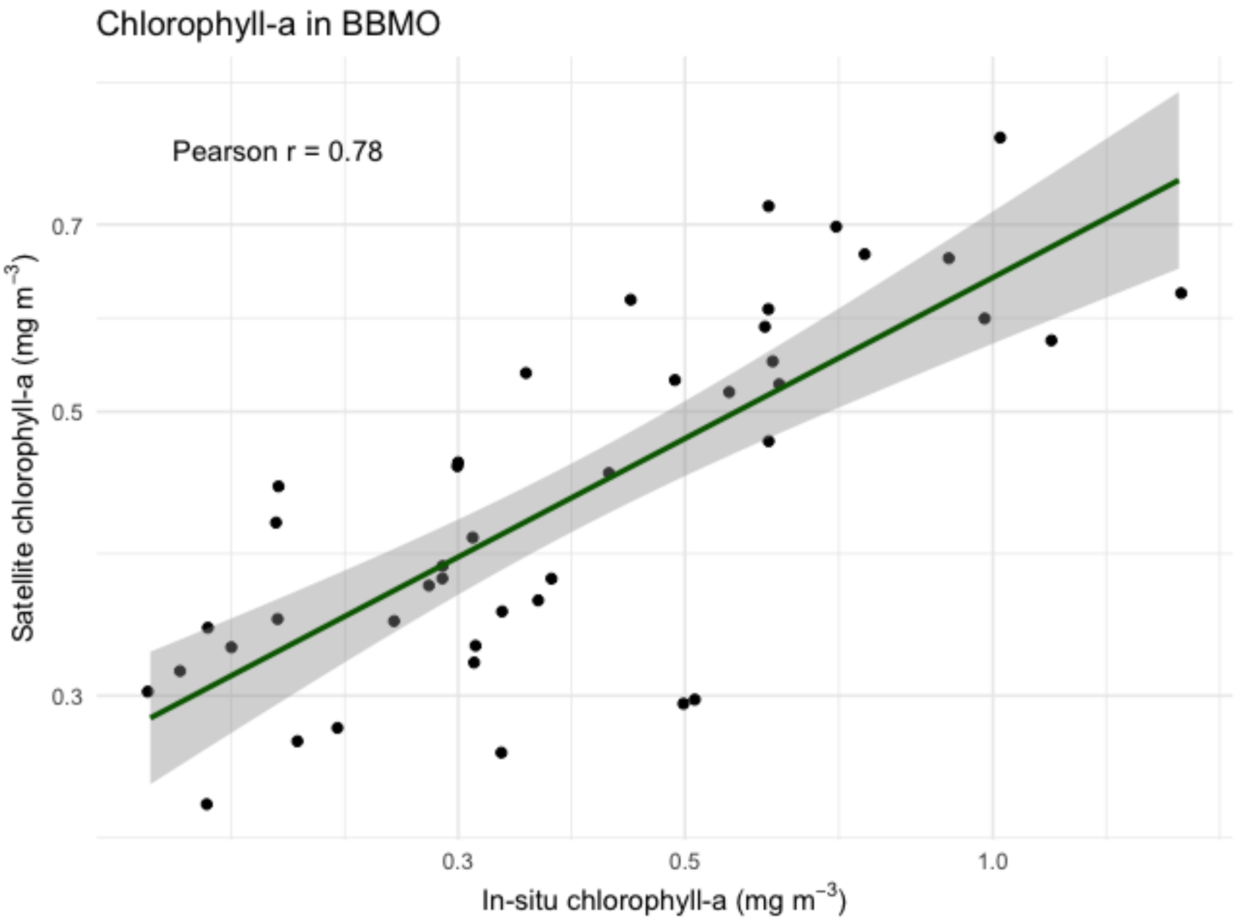

Figure S3. Correlation between satellite-derived and *in situ* chlorophyll *a* estimates at the Blanes Bay Microbial Observatory (BBMO). The correlation was statistically significant (Pearson's r= 0.78, p<0.01).

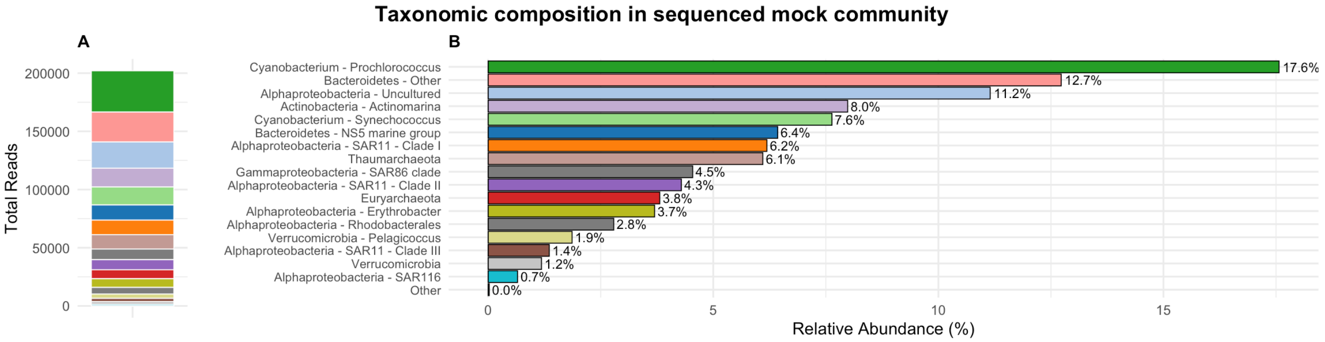

Figure S4. Taxonomic composition of the sequenced mock community. **(A)** Total number of reads assigned to each taxonomic group in the mock community sample. Colors represent different taxonomic groups in the mock community (Table S1). **(B)** Relative abundance of taxonomic groups. Values shown as percentages of total reads in the sample.

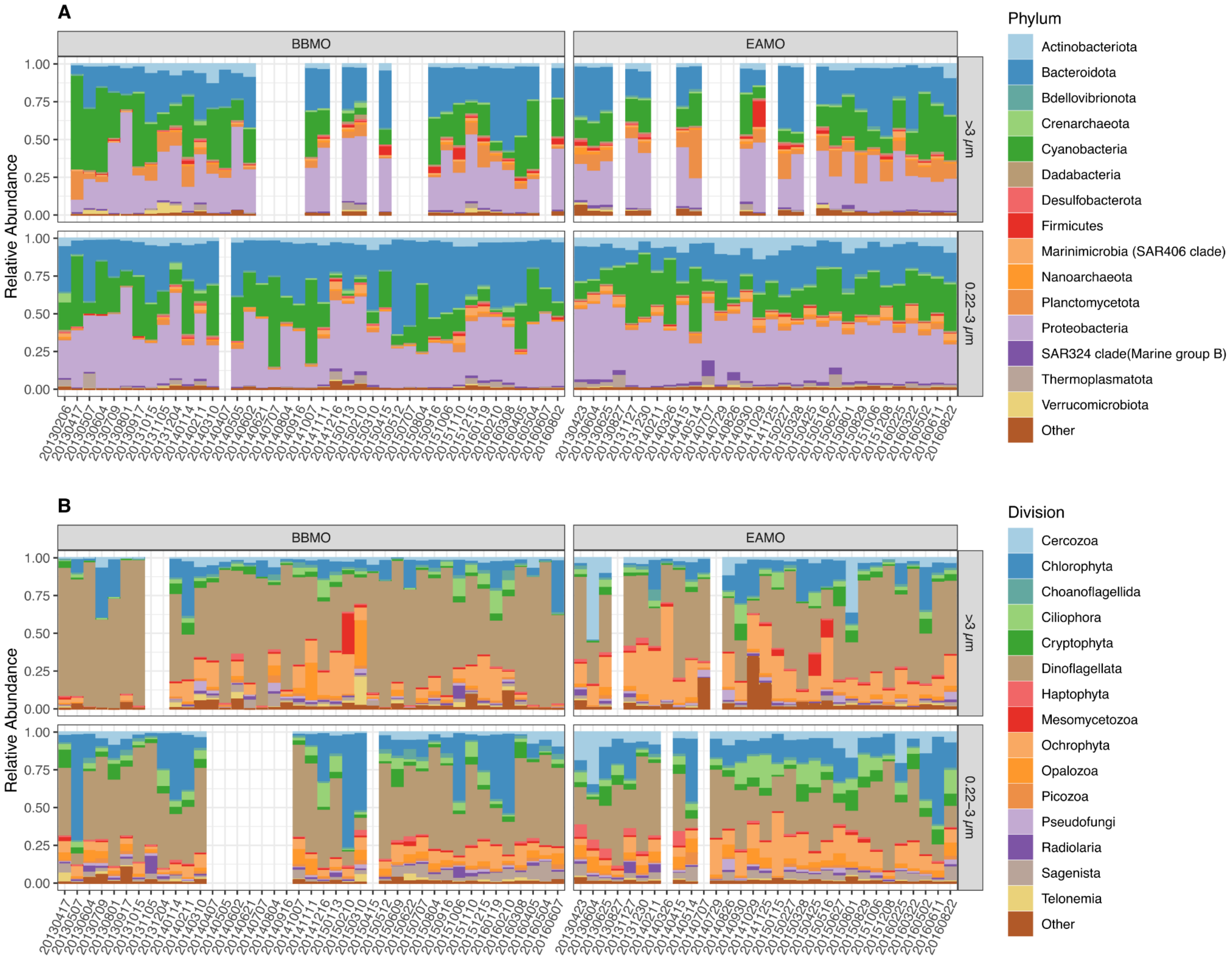

Figure S5. Taxonomic composition across samples of the **A)** prokaryotic and **B)** protist community in both size-fractions (0.22-3 μm and >3 μm) at the Blanes Bay Microbial Observatory (BBMO – Temperate site) and the Equatorial Atlantic Microbial Observatory (EAMO – Tropical site). The empty columns are samples with sub-optimal sequencing.

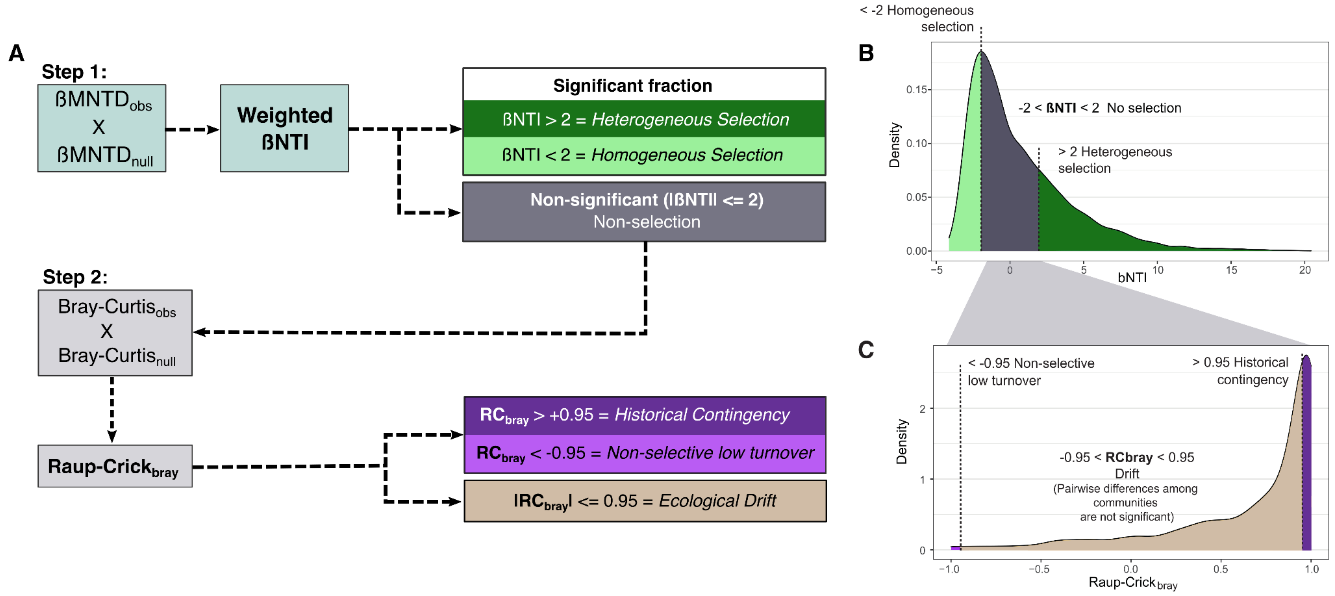

Figure S6. A) Summary of the analytical framework adapted from Stegen et al. 2013 to estimate ecological processes from probabilistic models using time-series data. The visualization of the model distributions **(B, C)** is adapted from (Gazulla et al., 2022). **B)** Distribution of βNTI distribution across all pairwise comparisons in the dataset. Absolute βNTI values greater than 2 indicate significant deviations from random phylogenetic turnover, suggesting the influence of either homogeneous or heterogeneous selection. The grey area highlights the range of nonsignificant βNTI values. To further differentiate whether drift or historical contingency are driving community turnover among these comparisons, we calculated the Bray–Curtis-based Raup–Crick metric (RC_{bray}). **C)** Distribution of RC_{bray} values for the subset of pairwise comparisons not structured by selection. RC_{bray} values between –0.95 and +0.95 suggest community assembly dominated by drift. Values > +0.95 or < –0.95 indicate that turnover is primarily shaped by historical contingency or rare non-selective processes, respectively.

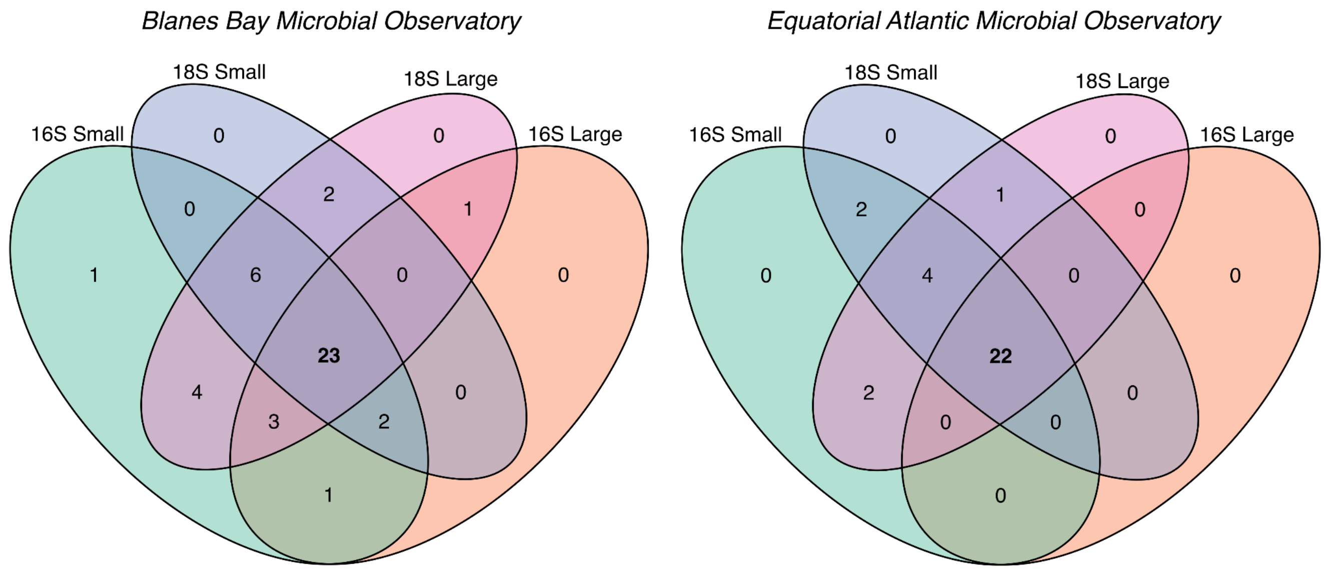

Figure S7. Venn's diagrams show the number of samples that had both 16S (prokaryotes) and 18S (protists) data, and "small" (0.22–3 µm) and "large" (>3 µm) size fractions. These samples were therefore retained for the network construction of BBMO (n=23) and EAMO (n=22).

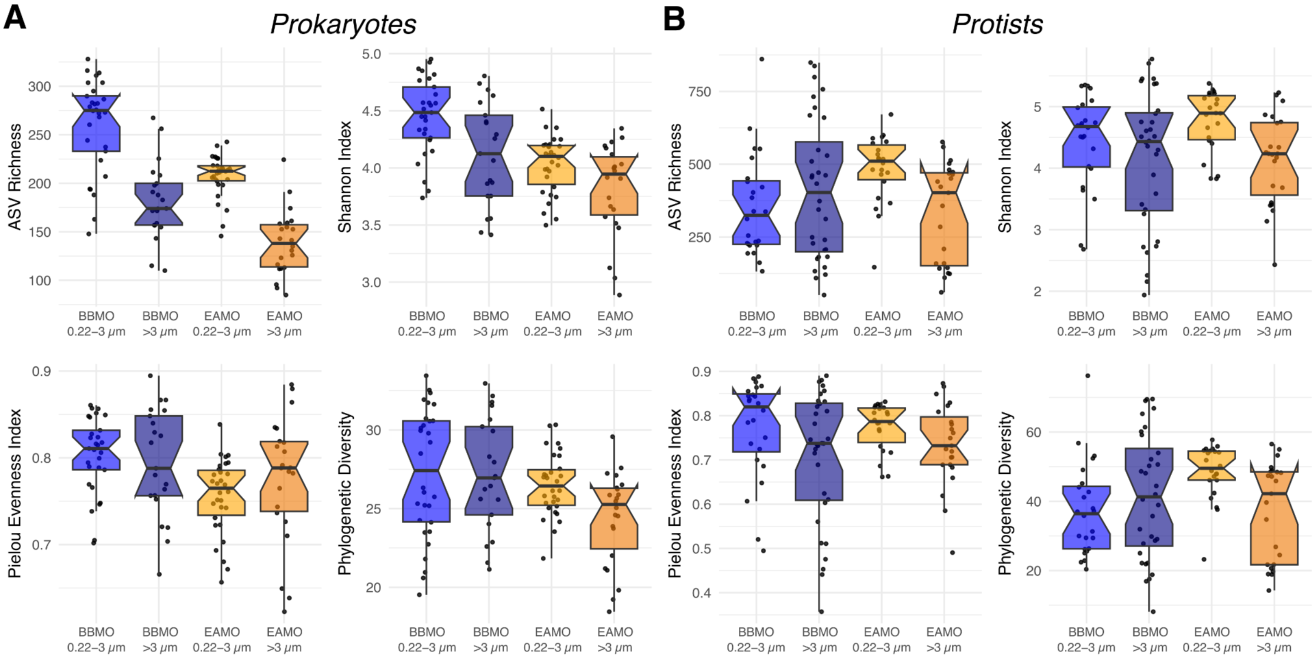

Figure S8. Diversity metrics showing the ASV richness, Pielou's evenness index, Shannon diversity index, and phylogenetic diversity, for each site and fraction.

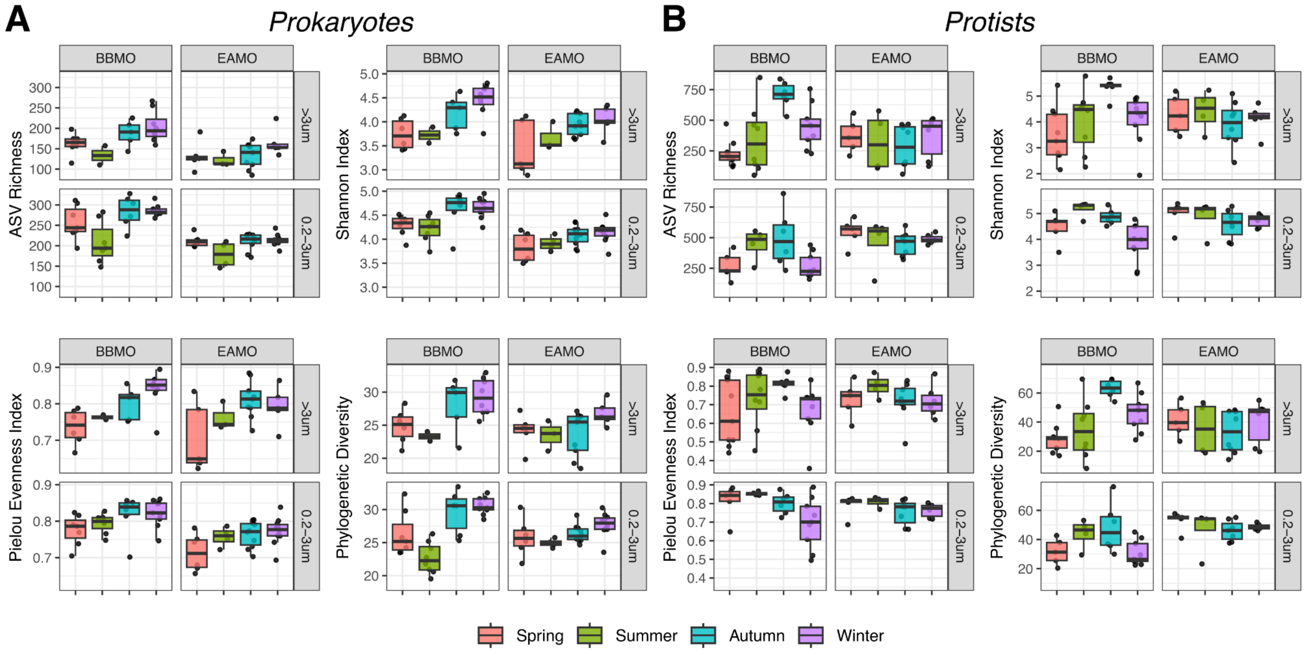

Figure S9. Seasonal differences in diversity metrics of **(A)** prokaryotes and **(B)** protists in the Blanes Bay Microbial Observatory (BBMO) and the Equatorial Atlantic Microbial Observatory (EAMO). The seasons were astronomically defined based on the dates in the Northern and the Southern hemispheres, respectively.

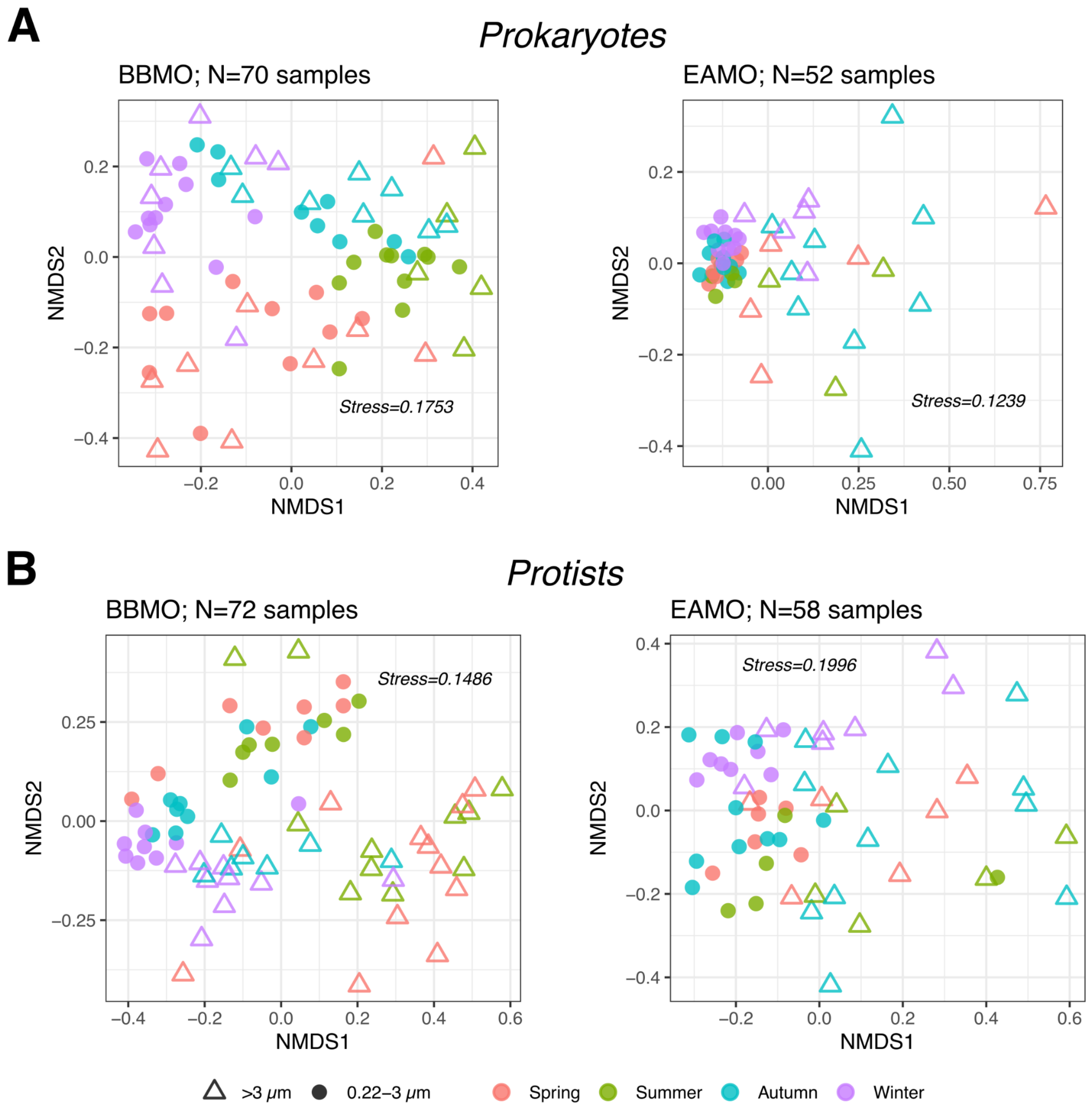

114

115 **Figure S10.** Nonmetric multidimensional scaling (NMDS) based on the Bray-Curtis dissimilarities among

116 prokaryotic and eukaryotic samples – labeled by seasons (colors) and size-fraction (shapes).

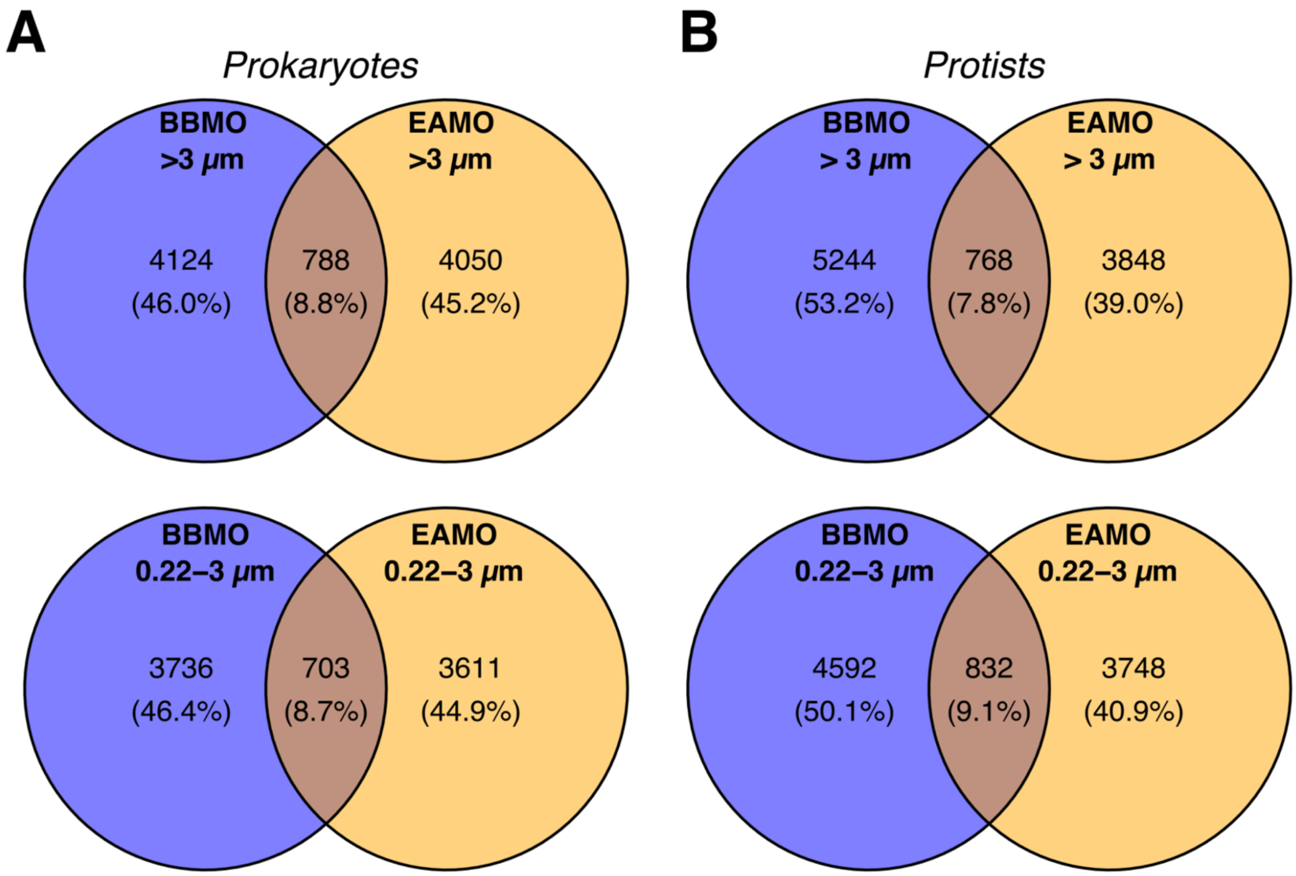

Figure S11. Venn's plots with the number of shared and unique **(A)** prokaryotic and **(B)** protist ASVs in each size-fraction between sites.

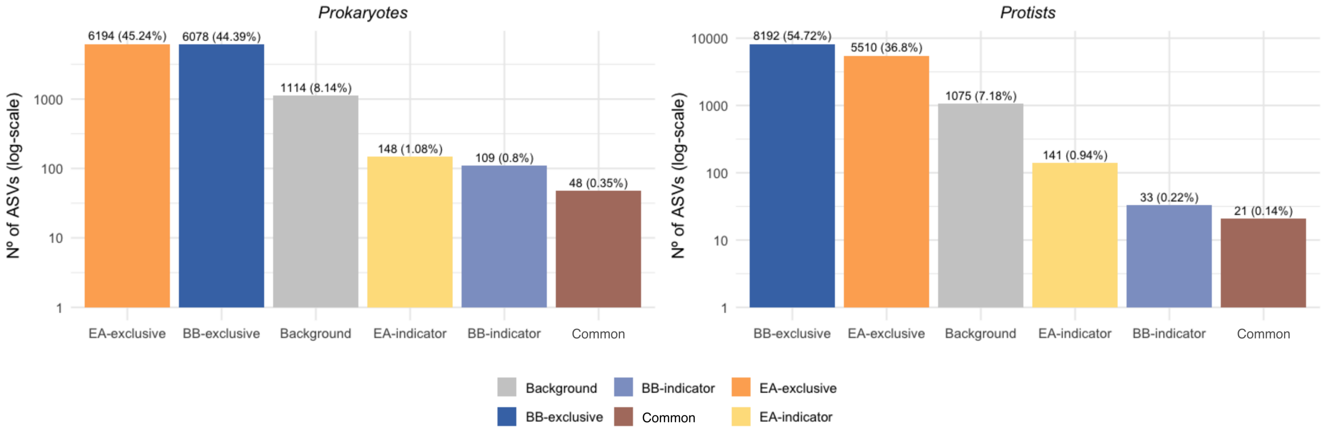

Figure S12. Number of ASVs within each category, as described in the methods. The percentage of total ASVs is indicated above each bar. Bars are sorted in decreasing order by number of ASVs. BB – Blanes Bay Microbial Observatory; EA – Equatorial Atlantic Microbial Observatory.

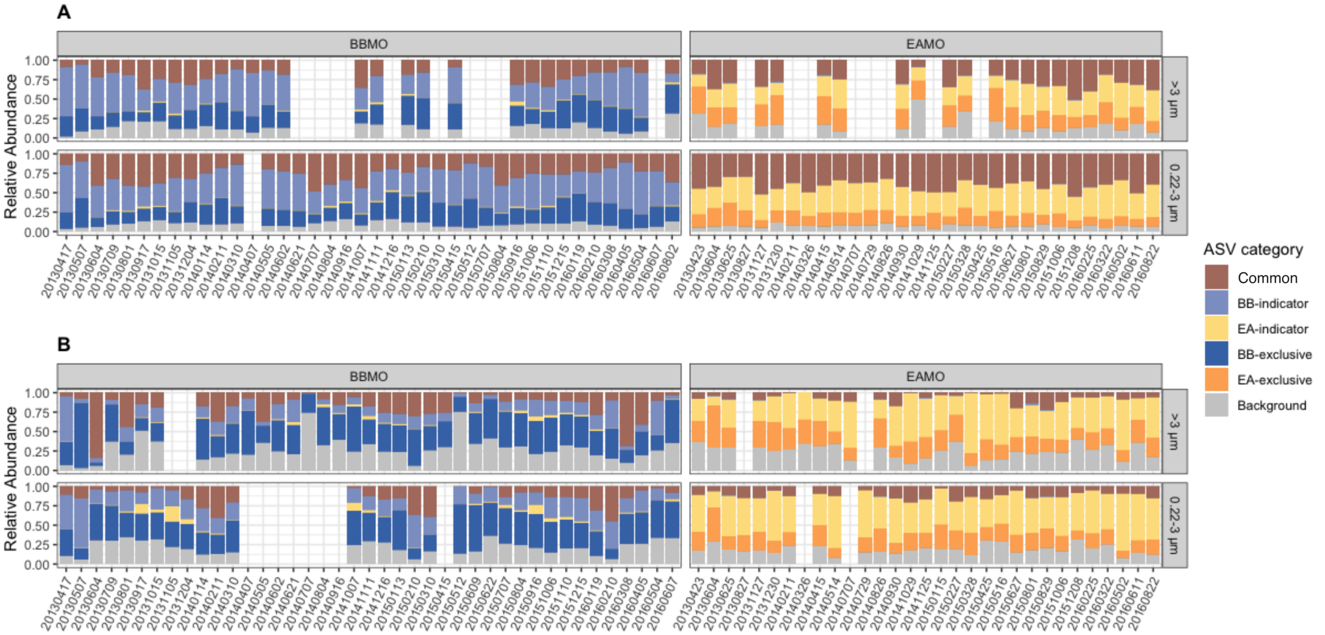

Figure S13. Relative abundance of the ASV categories of the **(A)** prokaryotic and **(B)** protist communities across samples. BBMO – Blanes Bay Microbial Observatory; EAMO – Equatorial Atlantic Microbial Observatory. The empty columns are samples with sub-optimal sequencing.

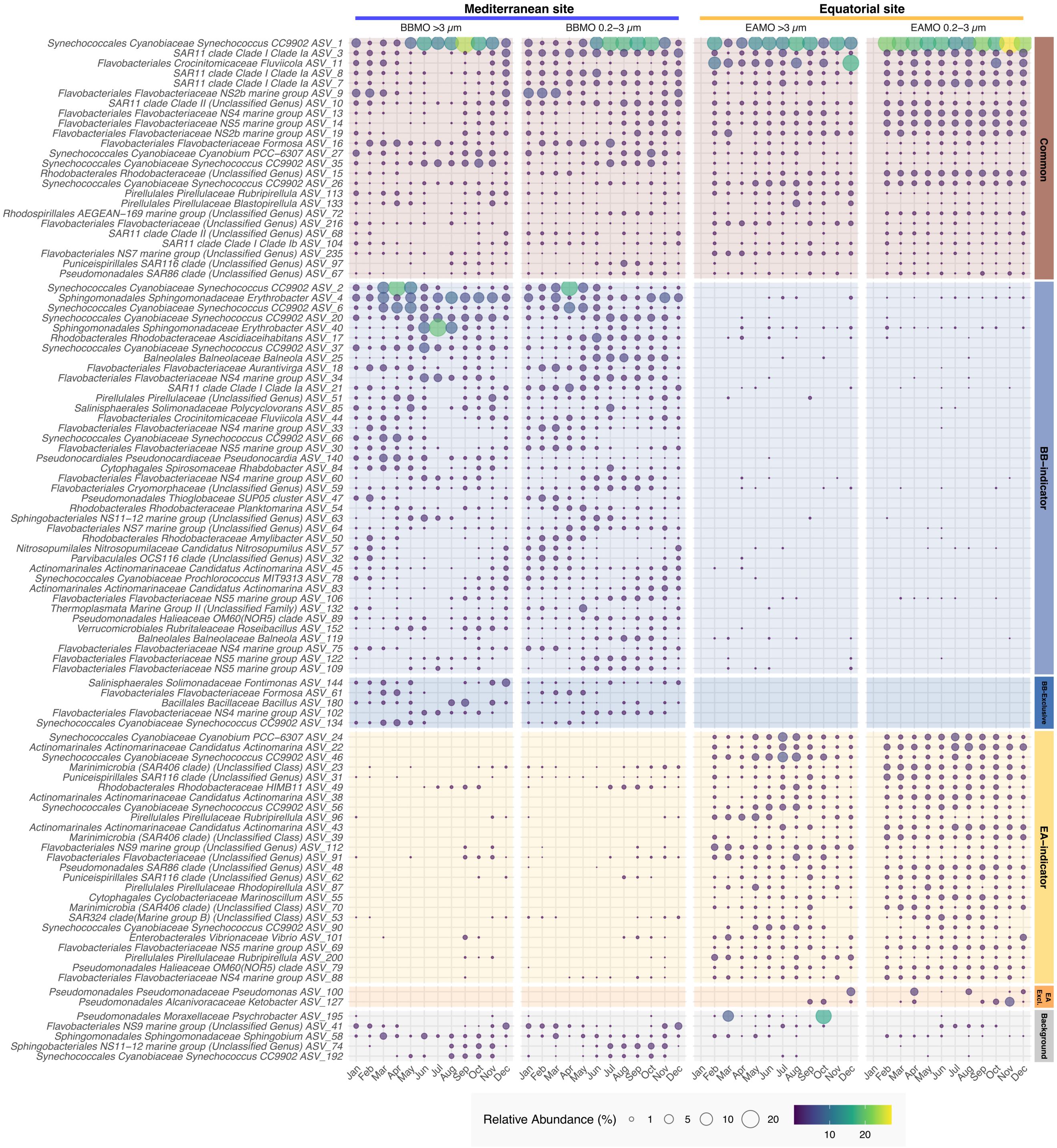

Figure S14. The monthly average relative abundance of the 100 most abundant prokaryotic ASVs classified as common, BB-indicators, EA-indicators, BBMO-exclusive, EAMO-exclusive, or background. BB = BBMO, EA = EAMO. The BBMO-exclusive and EAMO-exclusive categories refer to the ASVs which were unique to the station, but not statistically determined as an indicator.

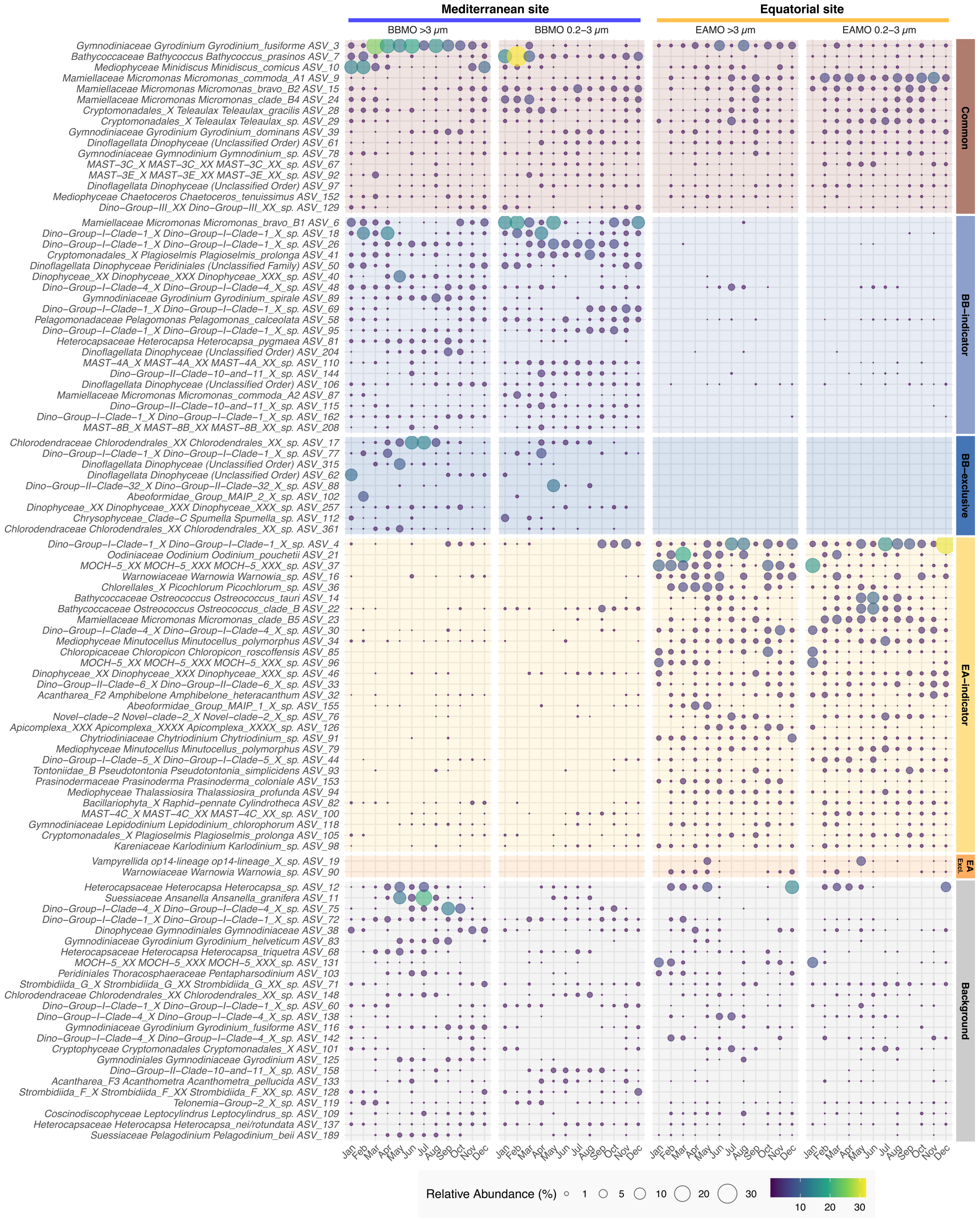

Figure S15. The monthly average relative abundance of the 100 most abundant protist ASVs classified as common, BB-indicators, EA-indicators, BBMO-exclusive, EAMO-exclusive, or background. BB = BBMO, EA =

EAMO. The BBMO-exclusive and EAMO-exclusive categories refer to the ASVs, which were unique to the station, but not statistically determined as an indicator.

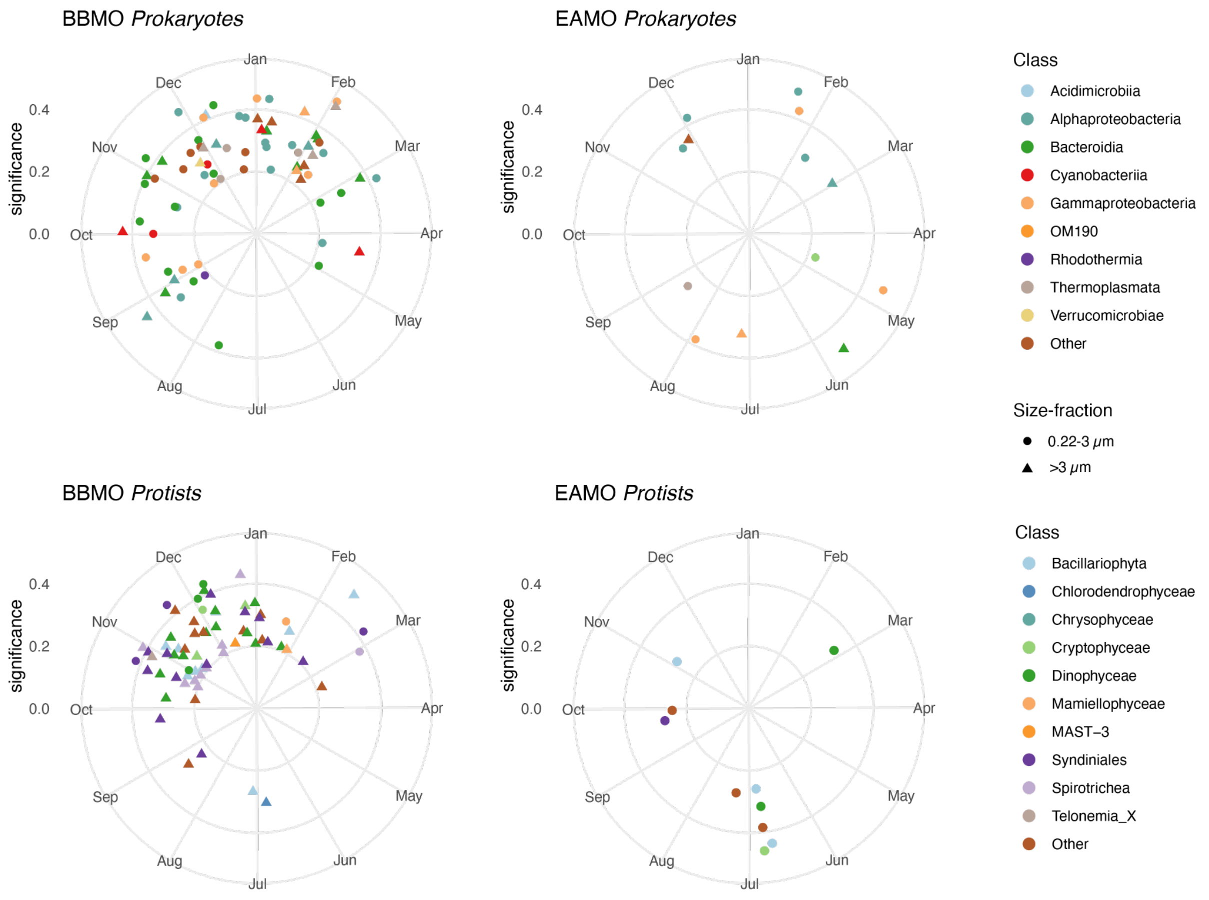

Figure S16. Polar plots representing the seasonal ASVs of the prokaryotic and protist communities. Different symbols indicate size fractions. ASVs are color-coded by taxonomic groups. Higher strength recurrence values represent stronger seasonal signals.

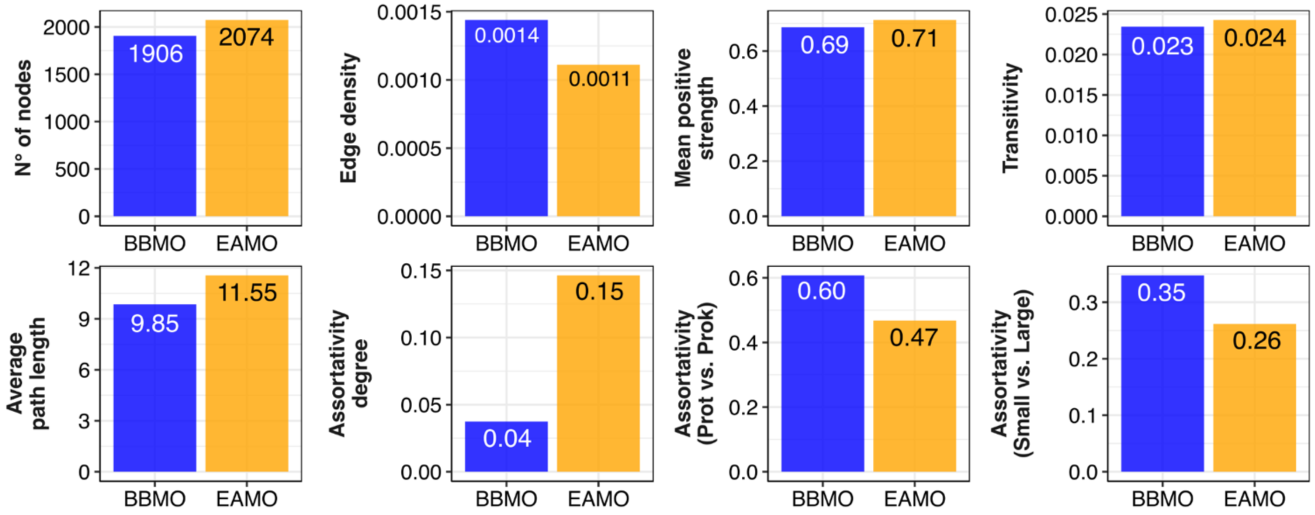

Figure S17. Bar plots showing the topological metrics of the Blanes Bay Microbial Observatory (BBMO) and the Equatorial Atlantic Microbial Observatory (EAMO) obtained from the static networks.

References

Alonso-Sáez, L., Balagué, V., Sà, E. L., Sánchez, O., González, J. M., Pinhassi, J., Massana, R., Pernthaler, J., Pedrós-Alió, C., & Gasol, J. M. (2007). Seasonality in bacterial diversity in north-west Mediterranean coastal waters: Assessment through clone libraries, fingerprinting and FISH. *FEMS Microbiology Ecology*, *60*(1), 98–112. https://doi.org/10.1111/j.1574-6941.2006.00276.x

Auladell, A., Barberán, A., Logares, R., Garcés, E., Gasol, J. M., & Ferrera, I. (2022). Seasonal niche differentiation among closely related marine bacteria. *The ISME Journal*, *16*(1), Article 1. https://doi.org/10.1038/s41396-021-01053-2

Catlett, D., Matson, P. G., Carlson, C. A., Wilbanks, E. G., Siegel, D. A., & Iglesias-Rodriguez, M. D. (2020). Evaluation of accuracy and precision in an amplicon sequencing workflow for marine protist communities. *Limnology and Oceanography: Methods*, *18*(1), 20–40. https://doi.org/10.1002/lom3.10343

Comeau, A. M., & Kwawukume, A. (2023). *Preparing multiplexed 16S/18S/ITS amplicons for the Illumina MiSeq v1*. Springer Science and Business Media LLC. https://doi.org/10.17504/protocols.io.4r3l277k3g1y/v1

Ferrera, I., Auladell, A., Balagué, V., Reñé, A., Garcés, E., Massana, R., & Gasol, J. M. (2024). Seasonal and interannual variability of the free-living and particle-associated bacteria of a coastal microbiome. *Environmental Microbiology Reports*, *16*(4). https://doi.org/10.1111/1758-2229.13299

Gazulla, C. R., Auladell, A., Ruiz-González, C., Junger, P. C., Royo-Llonch, M., Duarte, C. M., Gasol, J. M., Sánchez, O., & Ferrera, I. (2022). Global diversity and distribution of aerobic anoxygenic phototrophs in the tropical and subtropical oceans. *Environmental Microbiology*, *24*(5), 2222–2238. https://doi.org/10.1111/1462-2920.15835

Jing, M., Yang, W., Rao, L., Chen, J., Ding, X., Zhou, Y., Zhang, Q., Lu, K., & Zhu, J. (2024). Mechanisms of microbial coexistence in a patchy ecosystem: Differences in ecological niche overlap and species fitness between rhythmic and non-rhythmic species. *Water Research*, *256*, 121626. https://doi.org/10.1016/j.watres.2024.121626

Kirchman, D. (1992). Incorporation of thymidine and leucine in the subarctic Pacific application to estimating bacterial production. *Marine Ecology Progress Series*, *82*, 301–309. https://doi.org/10.3354/meps082301

Lambert, S., Tragin, M., Lozano, J.-C., Ghiglione, J.-F., Vaulot, D., Bouget, F.-Y., & Galand, P. E. (2019). Rhythmicity of coastal marine picoeukaryotes, bacteria and archaea despite irregular environmental perturbations. *The ISME Journal*, *13*(2), 388–401. https://doi.org/10.1038/s41396-018-0281-z

Lampe, R. H., Rabines, A. J., Ellman, B. A., Zheng, H., & Allen, A. E. (2025). Marine microbial mock communities for validating rRNA gene amplicon sequencing. *Microbiology Resource Announcements*. https://doi.org/10.1128/mra.00298-25

Marinchel, N., Marchesini, A., Nardi, D., Girardi, M., Casabianca, S., Vernesi, C., & Penna, A. (2023). Mock community experiments can inform on the reliability of eDNA metabarcoding data: A case study on marine phytoplankton. *Scientific Reports*, *13*(1), 20164. https://doi.org/10.1038/s41598-023-47462-5

Massana, R., DeLong, E. F., & Pedrós-Alió, C. (2000). A Few Cosmopolitan Phylotypes Dominate Planktonic Archaeal Assemblages in Widely Different Oceanic Provinces. *Applied and Environmental Microbiology*, *66*(5), 1777–1787. https://doi.org/10.1128/aem.66.5.1777-1787.2000

Parada, A. E., Needham, D. M., & Fuhrman, J. A. (2016). Every base matters: Assessing small subunit rRNA primers for marine microbiomes with mock communities, time series and global field samples: Primers for marine microbiome studies. *Environmental Microbiology*, *18*(5), 1403–1414. https://doi.org/10.1111/1462-2920.13023

Ruf, T. (1999). The Lomb-Scargle Periodogram in Biological Rhythm Research: Analysis of Incomplete and Unequally Spaced Time-Series. *Biological Rhythm Research*, *30*(2), 178–201. https://doi.org/10.1076/brhm.30.2.178.1422

Smith, D. C., & Azam, F. (1992). A simple, economical method for measuring bacterial protein synthesis rates in seawater using 3H-leucine. *Marine Microbial Food Webs*, *6*(2), 107–114.

Zhao, Z., Zhang, L., Zhang, G., Gao, H., Chen, X., Li, L., & Ju, F. (2023). Hydrodynamic and anthropogenic disturbances co-shape microbiota rhythmicity and community assembly within intertidal groundwater-surface water continuum. *Water Research*, *242*, 120236. https://doi.org/10.1016/j.watres.2023.120236
